# Relative survival among cancer survivors enrolled in private cancer insurance in Japan, using the internal insurance-enrolled population as the reference

**DOI:** 10.1007/s10147-025-02871-6

**Published:** 2025-09-09

**Authors:** Makoto Hiraoka, Hayaka Uekusa, Akira Okada, Reiko Inoue, Kayo Ikeda-Kurakawa, Yusuke Otsuka, Daisuke Namiki, Ryuichi Yamamoto, Toshimasa Yamauchi, Masaomi Nangaku, Kazuhiko Ohe, Satoko Yamaguchi, Takashi Kadowaki

**Affiliations:** 1 Department of Prevention of Diabetes and Lifestyle-Related Diseases, Graduate School of Medicine, The University of Tokyo, Tokyo, 113-8655 Japan; 2 Asahi Mutual Life Insurance Company, Tokyo, Japan; 3 Medical Information System Development Center, Tokyo, Japan; 4 Department of Diabetes and Metabolic Diseases, Graduate School of Medicine, The University of Tokyo, Tokyo, Japan; 5 Division of Nephrology and Endocrinology, Graduate School of Medicine, The University of Tokyo, Tokyo, Japan; 6 Department of Healthcare Information Management, The University of Tokyo Hospital, Tokyo, Japan; 7 Department of Bio-Medical Informatics, Graduate School of Medicine, The University of Tokyo, Tokyo, Japan; 8 Toranomon Hospital, 2-2-2, Toranomon, Minato-Ku, Tokyo, 105-8470 Japan

**Keywords:** Cancer survivors, Relative survival, Conditional relative survival, Private cancer insurance

## Abstract

**Background:**

Limited data are available on relative survival (RS) among cancer survivors enrolled in private cancer insurance in Japan. Additionally, the incidence of second primary cancers or recurrences, as applicable, after a certain period remains unclear.

**Methods:**

We analyzed 8,846 cancer survivors, including carcinoma in situ, aged 15–79 years, enrolled in private cancer insurance between April 2005 and September 2021, and diagnosed before April 2022. Using the entire insurance-enrolled population as the reference, we estimated sex- and age group-specific RS, conditional RS (CRS), and age-standardized RS (ASR) by cancer type. The cumulative incidence of second primary cancers or recurrences, as applicable, was calculated among cancer-free 3-, 4-, and 5-year survivors.

**Results:**

Median ages at first diagnosis were 61.5 years for males and 55.0 years for females. Over median follow-up of 3.40 years, 1,772 deaths (45.4 per 1,000 person-years) occurred. The 5-year RS declined with age: 81.8% for males and 94.8% for females aged 15–39, but 68.5% and 71.8% for those aged 70–79. The 5-year CRS increased with time since diagnosis, exceeding 90% among 5-year survivors in all groups except males aged 70–79. Liver cancer survivors had the highest incidence of second primary cancers or recurrences, predominantly due to recurrences, even after 5 cancer-free years.

**Conclusion:**

We estimated sex- and age group-specific RS, CRS, and ASR by cancer type, and the incidence of second primary cancers or recurrences, using a database of private cancer insurance policyholders, though the findings may not be generalizable to the national population.

**Supplementary Information:**

The online version contains supplementary material available at 10.1007/s10147-025-02871-6.

## Introduction

Population aging, improvements in early detection, and advances in cancer therapies have led to an increase in the number of cancer survivors worldwide [1]. Cancer survivors account for approximately 5% of the total population in the United States and Europe [2, 3]. In Japan, the most aged country in the world, cancer is the leading cause of death, accounting for one-third of all deaths, with a cumulative lifetime cancer risk of 65.5% for males and 51.2% for females as of 2019 [4]. National reports indicate that the 5-year relative survival (RS) for cancer patients improved from 53.2% among those diagnosed in 1993–1996 to 64.1% among those diagnosed in 2009–2011 [5].

Long-term RS and conditional relative survival (CRS), defined as the relative survival among those who have survived a certain period after diagnosis, have been described in detail based on population-based and hospital-based cancer registries in Japan [6, 7]. However, data remain limited for subpopulations such as individuals enrolled in private insurance, who may differ from the general population in terms of baseline health profiles and socioeconomic status. Furthermore, the proportion of cancer survivors who develop recurrences or second primary cancers after a certain period following their initial cancer diagnosis remains unclear. Large-scale insurance claims-based databases, which have become powerful tools in healthcare research, do not necessarily fully address this issue, as many cancer survivors leave or change jobs after their diagnosis, resulting in changes in insurance coverage that hinder long-term follow-up.

Cancer survivors face various long-term challenges, including prolonged initial treatment, recurrence, an increased risk of developing second primary cancers [8, 9], and financial burdens [10]. Even in countries with publicly funded universal healthcare coverage, including Japan, financial hardship is common among cancer survivors and their families [11–13]. Private medical insurance is widely used to cover copayments and other disease-associated costs in Japan, and the demand for private cancer insurance is increasing. One survey reported that, in 2022, 35.2% of individuals aged 18 to 79 were enrolled in private insurance that provides benefits in the event of cancer [14]. Another survey reported that households with higher annual income were more likely to be covered by private insurance [15], suggesting that individuals enrolled in private insurance may have higher socioeconomic status.

Here, we utilized a database of individuals enrolled in private cancer insurance policies. These policies not only offer benefits at initial cancer diagnosis but also provide additional payments for continued treatment, recurrence, or second primary cancers. These benefits make it less likely for cancer survivors to withdraw from their policies, thereby allowing for long-term follow-up. We investigated sex- and age group-specific RS and CRS among cancer survivors diagnosed with their first cancer between the ages of 15 and 79 years, using the internal insurance-enrolled population as the reference. We also evaluated the incidence of second primary cancers or recurrences, as applicable, among individuals who did not receive a second payment within 3, 4, or 5 years after the first payment.

## Patients and Methods

### Data sources and study population

The policyholder database of Asahi Mutual Life Insurance Company was processed into Anonymized Personal Information in accordance with the Act on the Protection of Personal Information by Fair and safe use of Anonymized Standardized Health Data of Japan (FAST-HDJ, Tokyo), after disclosing the required information on the company’s website. The anonymized database was provided to the Department of Prevention of Diabetes and Lifestyle-Related Diseases, the University of Tokyo, under a contract.

The database contains details on enrollment dates, insurance types, age at enrollment, sex, coverage termination dates and reasons (e.g., death, contract cancellation, and nonpayment), benefit payment dates, payment types and amounts, and disease codes for benefit payments. The classification of cancer sites into 11 categories was defined by the company based on its internal disease coding system for benefit payments: “stomach”, “colorectal”, “liver”, “lung”, “breast”, “uterus”, “ovary”, “prostate/testis”, “leukemia”, “thyroid”, and “others”. The corresponding International Classification of Diseases, Tenth Revision (ICD-10) codes are listed in Supplementary Table 1. In the company’s internal coding system, all cancer cases, including carcinoma in situ (CIS), were included. However, ICD-10 codes were unavailable before May 2013, making it impossible to identify CIS cases during that period. Therefore, CIS cases were included in the main analysis to ensure longer follow-up, while the sensitivity analysis was restricted to cases with available ICD-10 codes, excluding CIS cases. Supplementary Table 2 provides a breakdown of ICD-10 codes for each disease category within the subgroup of cases with recorded ICD-10 codes (see Supplementary Methods for details).

Individuals with a history of cancer or conditions considered high risk for cancer, such as hepatitis B or C infection, liver cirrhosis, chronic hepatitis, chronic pancreatitis, asbestos-related lung disease, ulcerative colitis, Crohn’s disease, or connective tissue disease, were ineligible to enroll in the cancer insurance policy.

Individuals enrolled in the cancer insurance policy are eligible for a lump-sum benefit if they are diagnosed with cancer (including CIS cases), cervical dysplasia, myelodysplastic syndrome, or myeloproliferative disease, provided the diagnosis is made at least 90 days after enrollment. Furthermore, they are eligible for a second payment two years after the first payment (or one year for policies issued after October 2, 2019) under any of the following circumstances: 1) they require continued treatment for the previously covered cancer, involving hospitalization at or after the time they become eligible; 2) they are diagnosed with a new lesion, that is a recurrence or metastasis of the previously covered cancer; or 3) they are newly diagnosed with a second primary cancer. Information regarding whether the second payment was for continuous treatment, recurrent cancer, or second primary cancer was unavailable. Since our primary aim was to evaluate the incidence of recurrent or second primary cancer among individuals who had completed treatment for their first cancer, we focused on those who had not received a second payment within three years of the first payment, to avoid including individuals undergoing treatment beyond two years. For these survivors, second payments were considered to reflect second primary cancers or recurrences, as applicable.

**Fig. 1:**
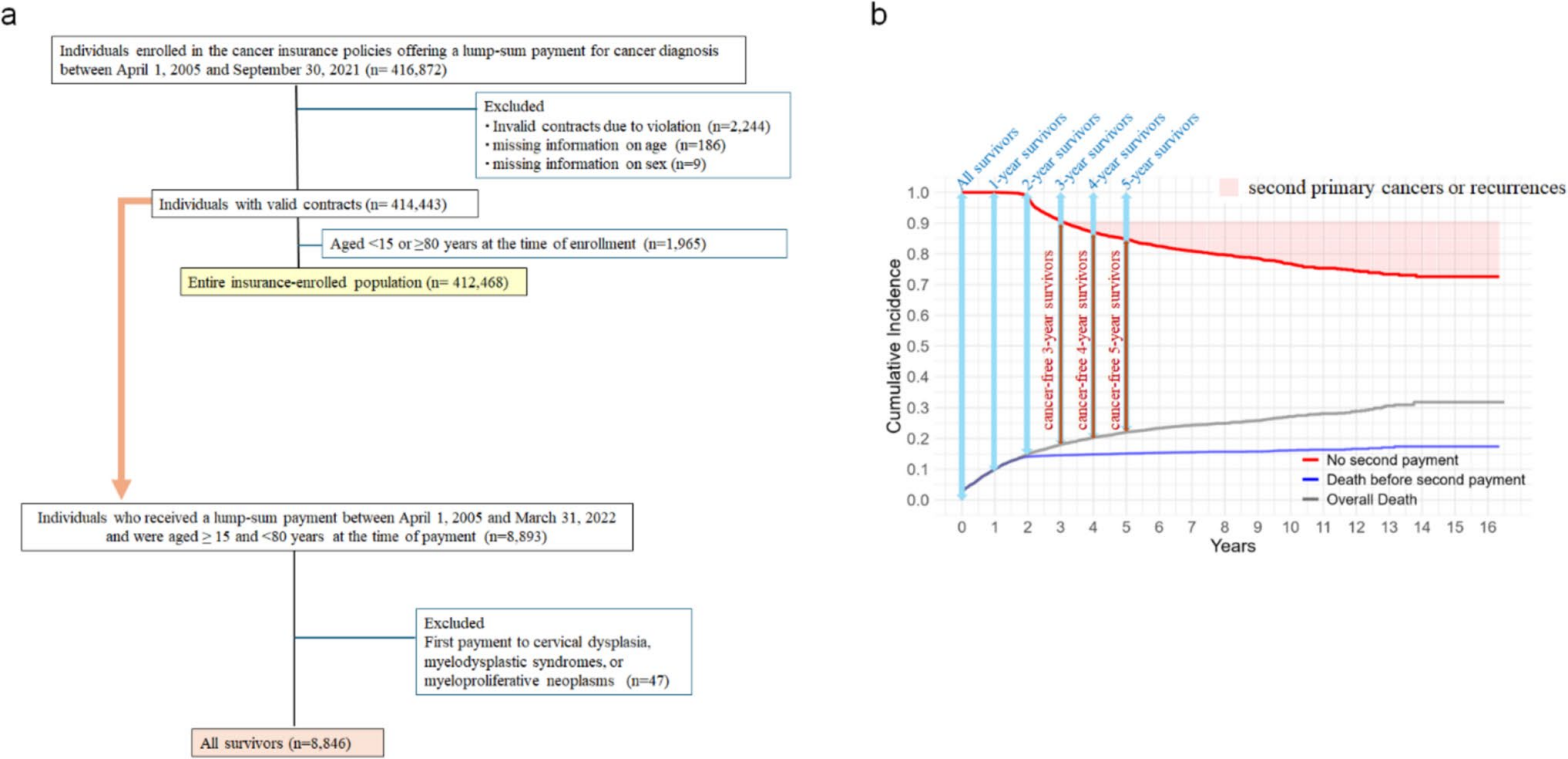
**a** Flowchart of the study. **b** Definition of the study population among “All survivors”

Among individuals enrolled in the insurance policy between April 1, 2005, and September 30, 2021, those with invalid contracts, such as breaches of the duty of disclosure, and those with missing information on sex or age were excluded. Individuals aged ≥ 15 and < 80 years at first enrollment were defined as the “Entire insurance-enrolled population”, which served as the reference group for estimating the incidence of first cancer, as well as RS (Fig. 1a). Individuals aged ≥ 80 years at the time of enrollment were excluded from the analysis, as they were ineligible to enroll in the insurance policy before April 2019.

Individuals who received their first benefit payment for a cancer diagnosis before April 1, 2022, and were aged ≥ 15 and < 80 years at the time of payment were selected. After excluding individuals who received the first benefit payment for cervical dysplasia, myelodysplastic syndrome, or myeloproliferative neoplasms, those who were diagnosed with cancer, including CIS cases, were defined as “All survivors” (Fig. 1a). Those with valid contracts *x* (1 to 5) years after the first payment were defined as “*x*-year survivors”. Among them, individuals who had not received a second payment within *x* (3 to 5) years were defined as “cancer-free *x*-year survivors” (Fig. 1b).

## Outcome measures

The outcomes of the study were as follows: (1) sex- and age group-specific RS and CRS, as well as age-standardized relative survival (ASR) by cancer type; and (2) the incidence of second primary cancers or recurrences, as applicable, among cancer-free survivors.

The dates of first cancer diagnosis and death were defined as the dates of the first payment and coverage termination due to death, respectively. If the payment occurred after the recorded date of death, the cancer diagnosis was considered to have occurred before death, and the date of cancer diagnosis was defined as the date of death. To estimate 5-year and 10-year RS of cancer survivors, we adopted an approach consistent with the Ederer II method [16], in which expected survival was calculated using the entire insurance-enrolled population, stratified by sex and age group. We used this internal reference group instead of national life tables to reduce potential selection bias, as individuals eligible for private insurance have better health profiles, which can affect background mortality. Cancer survivors and the entire insurance-enrolled population were followed from the date of first cancer diagnosis or enrollment, respectively, until death, coverage termination, or March 31, 2022, whichever occurred first (Supplementary Fig. 1). Age at the start of follow-up was categorized into five groups (15–39, 40–49, 50–59, 60 –69, and 70–79 years). The 5-year CRS, ASR by cancer type, and incidence of second primary cancers or recurrences, as applicable, were estimated as described in the Supplementary Methods. Because the Ederer II method includes cancer cases in the reference population, RS may be overestimated. To better approximate net survival, we conducted a sensitivity analysis in which individuals in the reference group were censored at the time of cancer diagnosis (see Supplementary Methods for details).

## Statistical analysis

All statistical analyses were performed using R version 4.2.3 (R Foundation for Statistical Computing, Vienna, Austria) and Easy R (EZR) [17]. For estimating survival and drawing survival curves, the *survfit()* function from the *survival* package in R was used. The cumulative incidence of cancer, with death before cancer treated as a competing risk, was estimated using the *cuminc()* function from the *cmprsk* package in R and the *summary.ci()* function in EZR, based on the cumulative incidence function [18].

Two-sided P values < 0.05 were considered significant. Further details are provided in the Supplementary Methods.

## Results

### Study population and overall deaths

A total of 412,468 individuals who enrolled in cancer insurance policies between April 1, 2005, and September 30, 2021, and were aged ≥ 15 and < 80 years at enrollment were defined as the “Entire insurance-enrolled population”, while 8,846 individuals aged ≥ 15 and < 80 years who were diagnosed with cancer before April 1, 2022, were defined as “All survivors” (Fig. 1). Characteristics of “All survivors” by sex and age group are shown in Table 1. The median age at first cancer diagnosis was 61.5 years (interquartile range [IQR], 53.0–68.0) for males and 55.0 years (IQR 44.0–66.0) for females, with 55.8% of the individuals being female. With a median follow-up of 3.40 years (39,063 person-years), 1772 deaths (45.4 deaths per 1000 person-years) were observed, including 1246 deaths occurring before the second payment. The overall death rate was 20.0%, and 13.1% received second payments. Characteristics of “All survivors” stratified by type of first cancer are presented in Supplementary Table 3, while those with recorded ICD-10 codes, excluding CIS cases, stratified by sex and age group are shown in Supplementary Table 4. Characteristics of “All survivors” categorized by death are summarized in Supplementary Table 5.

**Table 1:** Characteristics of “All survivors (diagnosed 2005–2022, including CIS cases)” by sex and age group

	All	Male						Female					
Age group	Total	Total	15–39	40–49	50–59	60–69	70–79	Total	15–39	40–49	50–59	60–69	70–79
n	8846	3912	246	481	973	1353	859	4934	771	1043	1150	1173	797
Age at first cancer diagnosis, median [IQR)]	59.0 [48.0, 67.0]	61.5 [53.0, 68.0]	34.0 [30.0, 37.0]	45.0 [43.0, 48.0]	55.0 [53.0, 58.0]	65.0 [62.0, 67.0]	73.0 [71.0, 76.0]	55.0 [44.0, 66.0]	34.0 [30.0, 37.0]	45.0 [42.0, 47.0]	54.0 [52.0, 57.0]	64.0 [62.0, 67.0]	73.0 [71.0, 76.0]
Sex, n(%)													
Female	4934 (55.8)	N/A	N/A	N/A	N/A	N/A	N/A	4934 (100.0)	771 (100.0)	1043 (100.0)	1150 (100.0)	1173 (100.0)	797 (100.0)
Type of first cancer, n(%)													
Stomach	813 (9.2)	500 (12.8)	21 (8.5)	50 (10.4)	121 (12.4)	191 (14.1)	117 (13.6)	313 (6.3)	20 (2.6)	35 (3.4)	67 (5.8)	100 (8.5)	91 (11.4)
Colorectal	1655 (18.7)	924 (23.6)	43 (17.5)	158 (32.8)	283 (29.1)	297 (22.0)	143 (16.6)	731 (14.8)	29 (3.8)	77 (7.4)	194 (16.9)	253 (21.6)	178 (22.3)
Liver	172 (1.9)	121 (3.1)	6 (2.4)	13 (2.7)	37 (3.8)	47 (3.5)	18 (2.1)	51 (1.0)	1 (0.1)	2 (0.2)	14 (1.2)	12 (1.0)	22 (2.8)
Lung	669 (7.6)	419 (10.7)	7 (2.8)	36 (7.5)	100 (10.3)	172 (12.7)	104 (12.1)	250 (5.1)	8 (1.0)	13 (1.2)	51 (4.4)	92 (7.8)	86 (10.8)
Thyroid	215 (2.4)	62 (1.6)	13 (5.3)	20 (4.2)	12 (1.2)	13 (1.0)	4 (0.5)	153 (3.1)	44 (5.7)	36 (3.5)	42 (3.7)	25 (2.1)	6 (0.8)
Leukemia	119 (1.3)	65 (1.7)	14 (5.7)	10 (2.1)	17 (1.7)	16 (1.2)	8 (0.9)	54 (1.1)	12 (1.6)	7 (0.7)	8 (0.7)	18 (1.5)	9 (1.1)
Prostate/testis	550 (6.2)	550 (14.1)	42 (17.1)	17 (3.5)	76 (7.8)	218 (16.1)	197 (22.9)	N/A	N/A	N/A	N/A	N/A	N/A
Breast	1447 (16.4)	8 (0.2)	0 (0.0)	0 (0.0)	2 (0.2)	3 (0.2)	3 (0.3)	1439 (29.2)	118 (15.3)	447 (42.9)	419 (36.4)	297 (25.3)	158 (19.8)
Uterus	1010 (11.4)	N/A	N/A	N/A	N/A	N/A	N/A	1010 (20.5)	433 (56.2)	306 (29.3)	156 (13.6)	86 (7.3)	29 (3.6)
Ovary	145 (1.6)	N/A	N/A	N/A	N/A	N/A	N/A	145 (2.9)	23 (3.0)	36 (3.5)	41 (3.6)	28 (2.4)	17 (2.1)
Others	2051 (23.2)	1263 (32.3)	100 (40.7)	177 (36.8)	325 (33.4)	396 (29.3)	265 (30.8)	788 (16.0)	83 (10.8)	84 (8.1)	158 (13.7)	262 (22.3)	201 (25.2)
Year of first cancer diagnosis, n(%)													
2005–2011	1522 (17.2)	688 (17.6)	77 (31.3)	94 (19.5)	228 (23.4)	224 (16.6)	65 (7.6)	834 (16.9)	174 (22.6)	209 (20.0)	223 (19.4)	175 (14.9)	53 (6.6)
2012–2017	3427 (38.7)	1498 (38.3)	105 (42.7)	201 (41.8)	366 (37.6)	552 (40.8)	274 (31.9)	1929 (39.1)	318 (41.2)	438 (42.0)	462 (40.2)	448 (38.2)	263 (33.0)
2018–2022	3897 (44.1)	1726 (44.1)	64 (26.0)	186 (38.7)	379 (39.0)	577 (42.6)	520 (60.5)	2171 (44.0)	279 (36.2)	396 (38.0)	465 (40.4)	550 (46.9)	481 (60.4)
Second payment, n(%)	1159 (13.1)	579 (14.8)	35 (14.2)	57 (11.9)	154 (15.8)	234 (17.3)	99 (11.5)	580 (11.8)	66 (8.6)	123 (11.8)	131 (11.4)	179 (15.3)	81 (10.2)
Death before second payment, n(%)	1246 (14.1)	756 (19.3)	33 (13.4)	75 (15.6)	173 (17.8)	267 (19.7)	208 (24.2)	490 (9.9)	23 (3.0)	48 (4.6)	116 (10.1)	161 (13.7)	142 (17.8)
Overall death, n(%)	1772 (20.0)	1019 (26.0)	45 (18.3)	99 (20.6)	249 (25.6)	373 (27.6)	253 (29.5)	753 (15.3)	42 (5.4)	102 (9.8)	172 (15.0)	252 (21.5)	185 (23.2)
Follow-up years, median (IQR)	3.40 [1.34, 6.76]	3.02 [1.04, 6.14]	5.80 [2.20, 9.51]	3.70 [1.36, 7.39]	3.34 [1.20, 7.07]	2.86 [0.99, 6.01]	2.20 [0.73, 4.16]	3.76 [1.67, 7.20]	5.19 [2.35, 8.95]	4.73 [2.33, 8.05]	4.05 [1.73, 7.84]	3.32 [1.44, 6.09]	2.32 [0.86, 4.26]

*CIS* carcinoma in situ, *IQR* interquartile range, *N/A* not applicable

### Sex- and age group-specific RS using the entire insurance-enrolled population as the reference

**Fig. 2:**
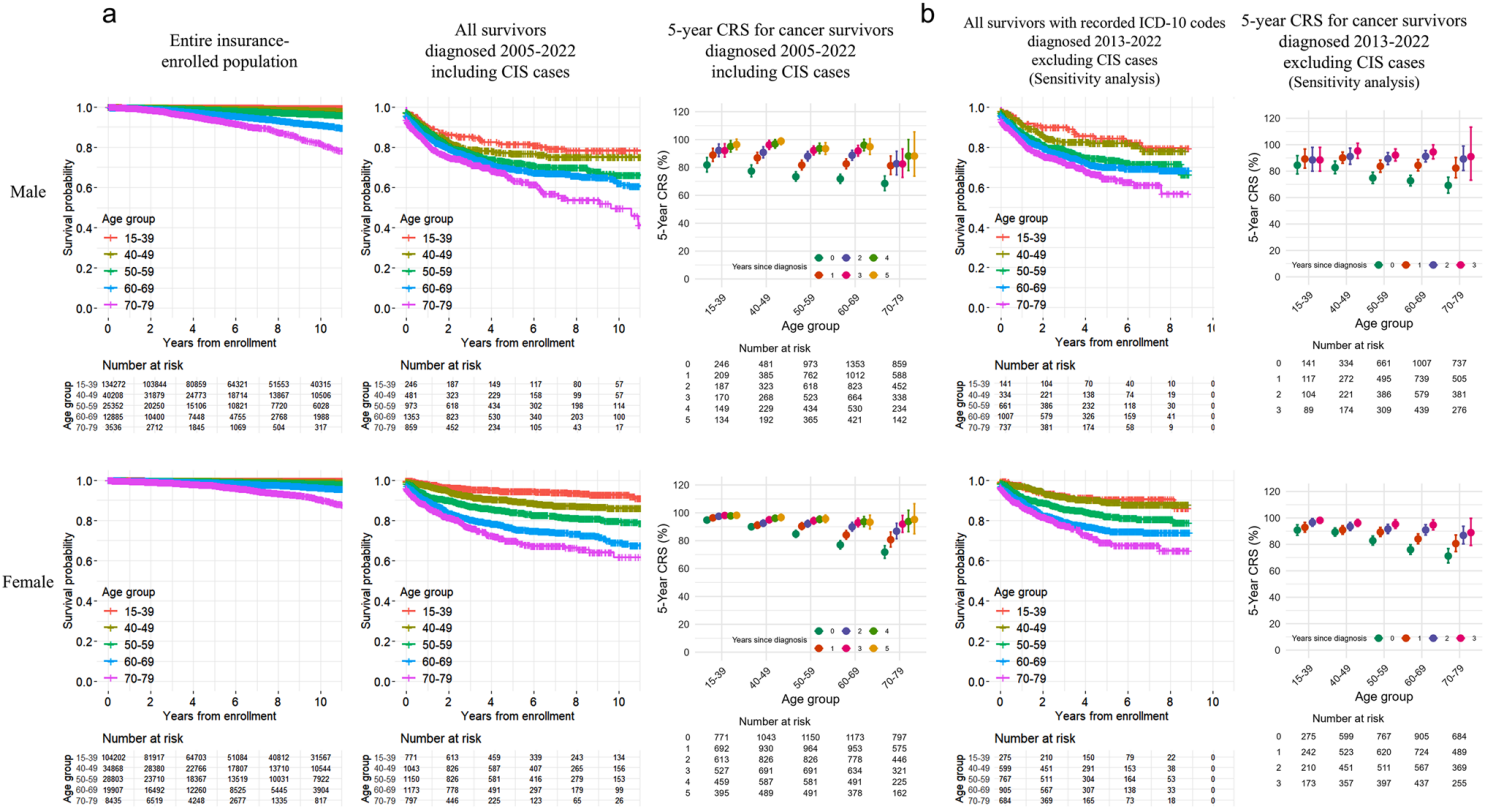
**a** Survival curves of “Entire insurance-enrolled population”, “All survivors (including CIS cases)” and the 5-year conditional relative survival (CRS) of “All survivors (including CIS cases)”, categorized by sex and age group **b** Survival curve and 5-year CRS of “All survivors with recorded ICD-10 codes (excluding CIS cases)” in the sensitivity analysis. Error bars indicate 95% confidence intervals. *CIS* carcinoma in situ, *CRS* conditional relative survival

Because individuals with a history of cancer or conditions considered high risk for cancer were ineligible to enroll in cancer insurance policies, the study population likely had better survival than the general population. To evaluate this difference, the cohort survival data of the entire insurance-enrolled population, along with the corresponding national statistics [19], are presented in Supplementary Table 6 and Supplementary Fig. 2, demonstrating higher survival in the study population. Sex- and age group-specific 5-year and 10-year RS, as well as 5-year CRS for cancer survivors are shown in Table 2, and survival curves for “All survivors” and “Entire insurance-enrolled population” along with the 5-year CRS for cancer survivors are shown in Fig. 2. The RS declined with age. The 5-year CRS increased with time since the first cancer diagnosis and exceeded 90% among 5-year survivors in all groups except for males aged 70–79. A sensitivity analysis excluding CIS cases was conducted using only those with recorded ICD-10 codes, yielding similar results. RS was lower when individuals were censored at first cancer diagnosis in the reference population, particularly among older survivors (Supplementary Table 7 and Supplementary Fig. 3).

**Table 2:** Sex- and age group-specific 5-year and 10-year relative survival and 5-year conditional relative survival among cancer survivors in the main analyses (including CIS cases) and sensitivity analyses (excluding CIS cases)

	Age group	Relative survival (95% CI)		5-year conditional relative survival (95% CI)				
		5-year RS	10-year RS	1-year survivors	2-year survivors	3-year survivors	4-year survivors	5-year survivors
Main analysis (diagnosed 2005–2022, including CIS cases)								
Male, by age group	15–39	81.8 (76.8–87.1)	78.9 (73.2–85.0)	88.9 (84.4–93.7)	92.2 (87.8–96.8)	92.1 (87.5–97.0)	95.1 (91.1–99.3)	96.4 (92.8–100.3)
	40–49	77.4 (73.3–81.8)	76.5 (72.0–81.3)	86.9 (83.1–90.8)	90.8 (86.9–94.8)	96.2 (93.0–99.4)	96.9 (93.9–100.0)	98.9 (96.4–101.1)
	50–59	73.4 (70.2–76.9)	68.7 (64.4–73.2)	81.7 (78.3–85.2)	88.1 (84.7–91.5)	92.2 (89.0–95.4)	93.6 (90.0–97.4)	93.5 (89.4–97.8)
	60–69	71.8 (68.6–75.1)	68.1 (63.2–73.3)	82.6 (79.1–86.1)	88.7 (85.2–92.3)	91.9 (88.2–95.8)	96.0 (92.0–100.2)	94.9 (89.4–100.6)
	70–79	68.5 (63.4–73.9)	60.4 (49.7–73.4)	81.3 (75.0–88.2)	82.7 (74.7–91.6)	82.5 (72.9–93.3)	88.2 (77.8–100.0)	88.2 (73.8–105.5)
Female, by age group	15–39	94.8 (93.0–96.6)	93.1 (90.8–95.5)	96.4 (94.8–98.0)	97.5 (96.0–99.0)	98.1 (96.6–99.5)	97.8 (96.0–99.6)	98.2 (96.6–99.9)
	40–49	90.0 (87.9–92.1)	87.0 (84.1–89.9)	91.1 (88.9–93.4)	92.5 (90.3–94.8)	95.0 (92.8–97.2)	96.1 (93.9–98.3)	96.7 (94.4–99.0)
	50–59	84.8 (82.4–87.4)	81.2 (78.0–84.6)	90.4 (88.0–92.9)	92.1 (89.8–94.6)	94.3 (91.9–96.7)	95.3 (93.0–97.7)	95.7 (93.0–98.4)
	60–69	77.0 (74.1–80.0)	71.8 (67.3–76.6)	84.1 (81.1–87.3)	89.9 (86.9–93.1)	93.0 (90.0–96.2)	93.6 (90.1–97.3)	93.3 (88.5–98.3)
	70–79	71.8 (67.4–76.4)	68.3 (60.0–77.8)	80.7 (75.5–86.2)	86.8 (81.4–92.5)	91.9 (86.0–98.1)	93.8 (86.5–101.8)	95.2 (85.0–106.5)
Sensitivity analysis (individuals with recorded ICD-10 codes, diagnosed 2013–2022, excluding CIS cases)								
Male with ICD-10 codes, by age group	15–39	84.5 (77.9–91.7)	N/D	89.2 (82.3–96.7)	88.5 (80.0–98.0)	88.5 (80.0–98.0)	N/D	N/D
	40–49	82.7 (78.1–87.6)	N/D	90.1 (86.0–94.4)	91.1 (85.2–97.4)	95.3 (89.7–101.1)	N/D	N/D
	50–59	74.8 (70.7–79.2)	N/D	83.7 (79.2–88.3)	89.4 (85.0–94.0)	92.1 (87.7–96.8)	N/D	N/D
	60–69	72.7 (68.9–76.8)	N/D	84.4 (80.2–88.8)	91.2 (87.0–95.6)	94.5 (89.3–99.9)	N/D	N/D
	70–79	69.2 (63.4–75.4)	N/D	82.3 (75.0–90.3)	89.2 (80.5–99.0)	91.0 (73.2–113.3)	N/D	N/D
Female with ICD-10 codes, by age group	15–39	90.8 (86.9–94.8)	N/D	92.9 (89.3–96.6)	96.6 (93.8–99.5)	98.2 (95.9–100.2)	N/D	N/D
	40–49	89.3 (86.3–92.4)	N/D	90.9 (87.7–94.1)	93.5 (90.5–96.6)	96.2 (93.6–98.9)	N/D	N/D
	50–59	82.8 (79.5–86.3)	N/D	89.3 (86.0–92.8)	91.6 (88.2–95.1)	95.3 (92.1–98.7)	N/D	N/D
	60–69	76.0 (72.5–79.7)	N/D	84.1 (80.5–87.9)	90.9 (87.1–94.9)	94.7 (91.0–98.6)	N/D	N/D
	70–79	71.2 (66.0–76.8)	N/D	80.6 (74.6–87.1)	86.8 (80.4–93.7)	88.9 (79.2–99.7)	N/D	N/D

*RS* relative survival, *CI *confidence interval, *CIS* carcinoma in situ, *ICD-10* International Classification of Diseases Tenth Revision, *N/D* not determined due to insufficient follow-up time

### ASR by cancer type

**Fig. 3:**
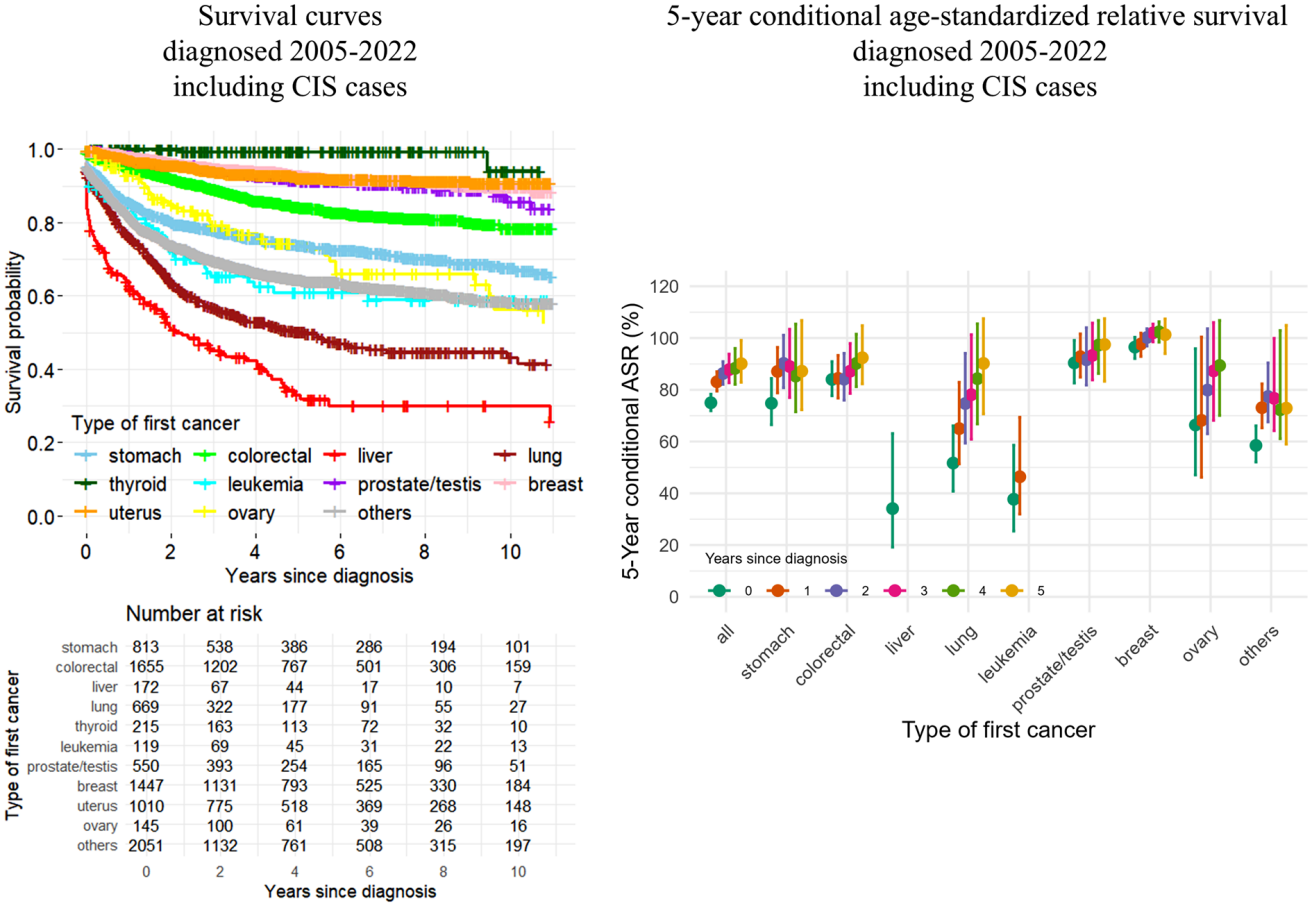
Survival curves and 5-year conditional age-standardized relative survival (ASR) by type of first cancer. Error bars indicate 95% confidence intervals. *CIS* carcinoma in situ

Figure 3 shows survival curves by cancer type, while Table 3 presents 5-year and 10-year crude survival, ASR and 5-year conditional ASR by cancer type. While the 5-year ASR was 34.1% for liver cancer, it was 96.5% for breast cancer. For most cancer types, the 5-year conditional ASR increased with time since the first cancer diagnosis. As a sensitivity analysis, cancer survivors with recorded ICD-10 codes were analyzed, excluding CIS cases (Supplementary Fig. 4 and Supplementary Table 8).

**Table 3:** 5-year and 10-year crude survival, age-standardized relative survival (ASR), and 5-year conditional ASR by cancer type (diagnosed 2005–2022, including CIS cases)

	Crude survival (95% CI)		ASR (95% CI)		5-year conditional ASR (95% CI)				
Group	5-year survival	10-year survival	5-year ASR	10-year ASR	1-year survivors	2-year survivors	3-year survivors	4-year survivors	5-year survivors
All	77.9 (76.9–78.9)	72.8 (71.5–74.1)	75.0 (71.9–78.3)	68.2 (62.6–75.1)	83.1 (79.5–87.0)	86.3 (82.1–90.9)	87.8 (82.7–93.8)	88.3 (82.1–96.0)	90.1 (82.9–99.1)
Stomach	73.8 (70.6–77.2)	67.7 (63.7–72.0)	74.8 (66.5–84.4)	65.3 (52.0–87.2)	87.1 (78.8–96.4)	90.2 (80.8–101.1)	89.0 (77.0–103.4)	85.3 (71.5–105.4)	87.2 (72.3–106.8)
Colorectal	84.3 (82.3–86.4)	78.5 (75.5–81.6)	84.0 (77.7–90.9)	77.8 (67.8–90.3)	84.4 (76.8–93.3)	84.1 (76.0–94.1)	87.2 (78.6–97.9)	90.2 (81.2–101.5)	92.4 (82.3–104.8)
Liver	33.0 (25.6–42.5)	30.0 (22.6–39.9)	34.1 (19.2–63.1)	N/D	N/D	N/D	N/D	N/D	N/D
Lung	50.2 (45.9–54.9)	43.2 (37.8–49.3)	51.7 (40.8–66.1)	46.7 (33.2–67.6)	65.0 (51.4–82.9)	74.7 (59.4–94.1)	78.1 (60.9–101.3)	84.3 (66.8–105.5)	90.2 (70.7–107.5)
Thyroid	99.4 (98.2–100.0)	94.1 (84.6–100.0)	N/D	N/D	N/D	N/D	N/D	N/D	N/D
Leukemia	61.0 (51.9–71.7)	58.9 (49.5–70.2)	37.7 (25.4–58.6)	38.3 (25.5–60.3)	46.4 (32.0–69.4)	N/D	N/D	N/D	N/D
Prostate/testis	91.2 (88.3–94.3)	85.7 (80.1–91.6)	90.4 (82.6–99.1)	88.2 (73.7–106.5)	92.8 (84.9–101.6)	91.6 (81.8–104.0)	93.3 (83.8–105.8)	97.3 (86.3–106.8)	97.5 (83.3–107.5)
Breast	92.9 (91.4–94.5)	89.6 (87.3–91.9)	96.5 (92.1–100.3)	98.0 (88.6–107.5)	97.7 (92.9–101.9)	100.2 (96.9–103.6)	102.0 (98.5–105.4)	102.4 (98.4–106.3)	101.3 (94.0–107.4)
Uterus	92.2 (90.3–94.1)	90.9 (88.6–93.2)	N/D	N/D	N/D	N/D	N/D	N/D	N/D
Ovary	74.4 (66.6–83.0)	56.3 (44.4–71.4)	66.4 (47.1–95.9)	N/D	68.2 (46.2–100.4)	79.9 (63.0–103.6)	87.3 (68.2–106.0)	89.4 (70.1–106.8)	N/D
Others	64.4 (62.1–66.8)	58.3 (55.4–61.2)	58.5 (52.1–66.1)	45.2 (36.9–69.2)	73.1 (65.3–82.3)	77.4 (67.6–90.4)	76.7 (64.2–99.9)	72.3 (61.1–102.9)	72.9 (59.0–104.9)

*CIS* carcinoma in situ, *ASR* age-standardized relative survival, *CI* confidence interval

*N/D* not determined: ASR could not be estimated for some cancer types due to an insufficient number of survivors in certain age groups with adequate follow-up time

### Cohort and period analyses

Cohort analyses for survivors diagnosed between 2005–2011 and 2012–2017, as well as period analyses for survivors followed between 2012–2016 and 2017–2021, were conducted (Supplementary Table 9 and Supplementary Fig. 5, see Supplementary Methods for details). The 5-year RS was higher in more recent years.

### Results of analyses for the incidence of second primary cancers or recurrences, as applicable

**Fig. 4:**
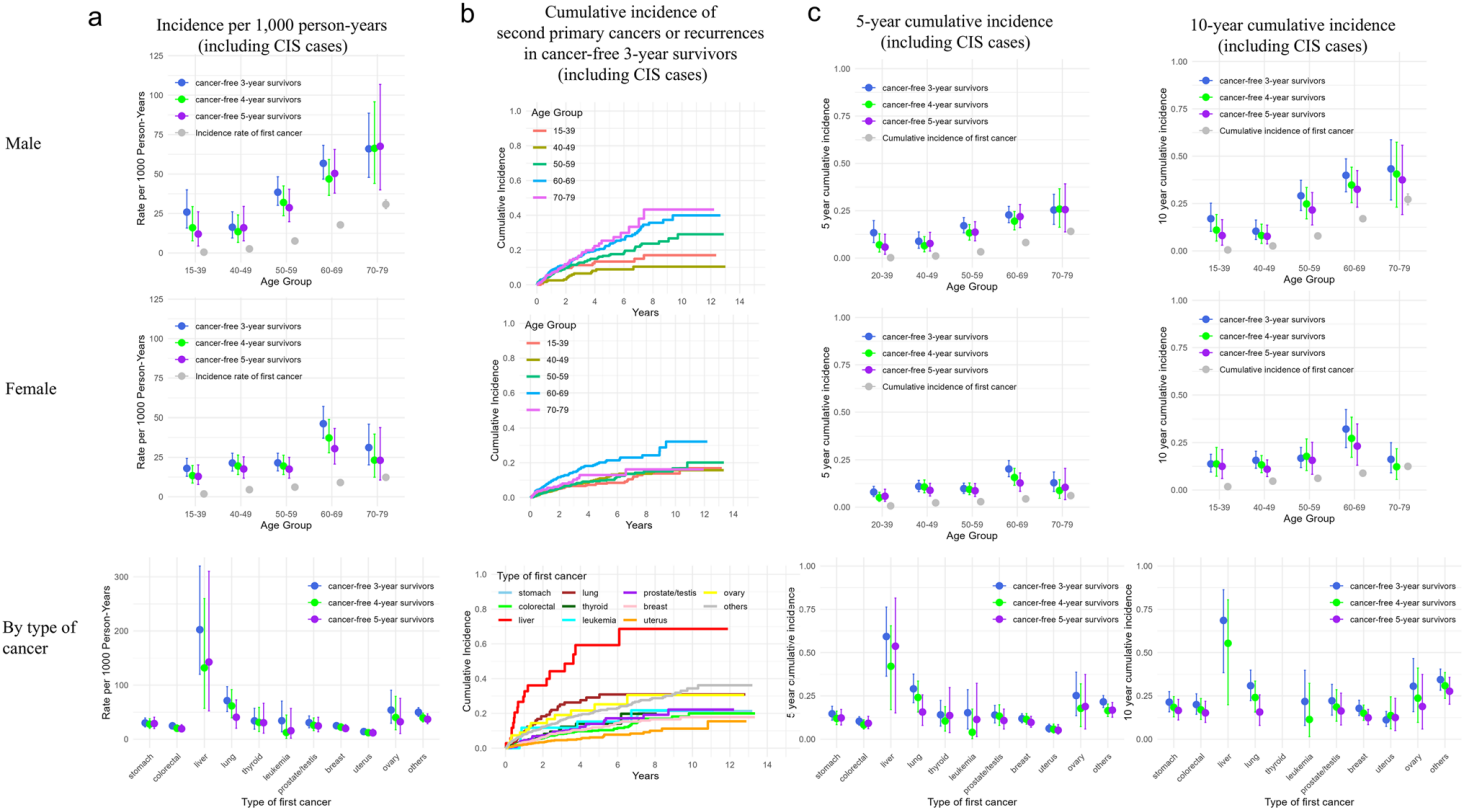
**a** Incidence rates of second primary cancers or recurrences, as applicable, per 1,000 person-years, categorized by sex and age group or by cancer type **b** Cumulative incidence curves of second primary cancers or recurrences, as applicable, among “cancer-free 3-year survivors” **c** 5-year and 10-year cumulative incidence of second primary cancers or recurrences, as applicable, categorized by sex and age group or by cancer type. Error bars indicate 95% confidence intervals. *CIS* carcinoma in situ

Next, we evaluated the incidence rates of second primary cancers or recurrences, as applicable, in cancer-free survivors. Incidence rates per 1000 person-years were calculated by sex and age group, as well as by cancer type (Fig. 4a and Supplementary Table 10). For reference, incidence rates of first cancers by sex and age group in the “Entire insurance-enrolled population” are also provided. The differences in incidence rates between first cancers and second primary cancers or recurrences, as applicable, were generally greater in younger individuals than in older individuals and in the “cancer-free 3-year survivors” than in the “cancer-free 5-year survivors”. Individuals who received the first payment for liver cancer showed the highest incidence rates of second primary cancers or recurrences, as applicable, even among the “cancer-free 5-year survivors”.

Cumulative incidence curves for second payment, death before second payment, and overall death in “All survivors”, as well as for first payment, death before first payment, and overall death in the “Entire insurance-enrolled population”, are shown in Supplementary Fig. 6. As the incidence rates of death before second payment were considerably high among cancer survivors, we estimated the cumulative incidence of second primary cancers or recurrences, as applicable, among cancer-free survivors, accounting for death as a competing risk. Cumulative incidence curves for second primary cancers or recurrences, as applicable, in “cancer-free 3-year survivors” are shown in Fig. 4b. The 5-year and 10-year cumulative incidence of second primary cancers or recurrences, as applicable, in each of the “cancer-free 3-, 4-, and 5-year survivors” by sex and age group are shown in Fig. 4c and Table 4. While the cumulative incidence of first cancer increased with age, the differences among age groups were less pronounced for second primary cancers or recurrences. Compared to the results based on the person-years method, differences between the cumulative incidence of first cancers and that of second primary cancers or recurrences, as applicable, were smaller in older age groups where the incidence of death before the second event was higher. Individuals who received the first payment for liver cancer had the highest cumulative incidence even among “cancer-free 5-year survivors”.

**Table 4:** Cumulative incidence of second primary cancers or recurrences, as applicable, accounting for death as a competing risk

5-year cumulative incidence (95% CI)					
	Group	Cancer-free 3-year survivors (first cancer diagnosed 2005–2019, including CIS cases)	Cancer-free 4-year survivors (first cancer diagnosed 2005–2018, including CIS cases)	Cancer-free 5-year survivors (first cancer diagnosed 2005–2017, including CIS cases)	First cancer
Male, by age group	15–39	0.134 (0.082–0.198)	0.070 (0.032–0.128)	0.058 (0.020–0.126)	0.002 (0.002–0.002)
	40–49	0.089 (0.052–0.138)	0.065 (0.033–0.113)	0.077 (0.037–0.136)	0.011 (0.010–0.012)
	50–59	0.171 (0.133–0.214)	0.133 (0.095–0.177)	0.137 (0.091–0.192)	0.033 (0.031–0.036)
	60–69	0.228 (0.187–0.273)	0.195 (0.149–0.246)	0.219 (0.161–0.283)	0.082 (0.076–0.088)
	70–79	0.254 (0.178–0.337)	0.259 (0.163–0.366)	0.256 (0.138–0.392)	0.141 (0.127–0.155)
Female, by age group	15–39	0.080 (0.056–0.110)	0.051 (0.031–0.079)	0.058 (0.031–0.095)	0.007 (0.007–0.008)
	40–49	0.110 (0.082–0.142)	0.107 (0.076–0.144)	0.089 (0.059–0.126)	0.023 (0.021–0.025)
	50–59	0.098 (0.073–0.127)	0.094 (0.066–0.128)	0.087 (0.057–0.125)	0.029 (0.027–0.032)
	60–69	0.202 (0.161–0.246)	0.157 (0.116–0.205)	0.128 (0.084–0.181)	0.044 (0.041–0.048)
	70–79	0.129 (0.083–0.186)	0.088 (0.047–0.145)	0.105 (0.040–0.205)	0.061 (0.055–0.068)
By type of first cancer	Stomach	0.147 (0.110–0.190)	0.120 (0.084–0.162)	0.123 (0.082–0.172)	N/A
	Colorectal	0.104 (0.081–0.130)	0.083 (0.059–0.112)	0.092 (0.061–0.132)	N/A
	Liver	0.593 (0.364–0.763)	0.421 (0.171–0.655)	0.537 (0.152–0.815)	N/A
	Lung	0.291 (0.211–0.376)	0.241 (0.157–0.336)	0.157 (0.080–0.256)	N/A
	Thyroid	0.141 (0.076–0.224)	0.105 (0.047–0.188)	0.137 (0.038–0.298)	N/A
	Leukemia	0.153 (0.059–0.286)	0.040 (0.003–0.174)	0.114 (0.015–0.323)	N/A
	Prostate/testis	0.140 (0.094–0.195)	0.133 (0.080–0.199)	0.108 (0.059–0.174)	N/A
	Breast	0.119 (0.095–0.147)	0.115 (0.087–0.148)	0.097 (0.069–0.132)	N/A
	Uterus	0.062 (0.041–0.087)	0.058 (0.036–0.087)	0.052 (0.030–0.084)	N/A
	Ovary	0.252 (0.136–0.387)	0.178 (0.073–0.320)	0.189 (0.059–0.374)	N/A
	Others	0.216 (0.183–0.252)	0.165 (0.131–0.203)	0.168 (0.127–0.212)	N/A

10-year cumulative incidence (95% CI)					
	Group	Cancer-free 3-year survivors	Cancer-free 4-year survivors	Cancer-free 5-year survivors	First cancer
Male, by age group	15–39	0.170 (0.103–0.252)	0.110 (0.052–0.192)	0.081 (0.030–0.165)	0.005 (0.005–0.006)
	40–49	0.104 (0.060–0.163)	0.081 (0.040–0.141)	0.077 (0.037–0.136)	0.026 (0.024–0.029)
	50–59	0.291 (0.213–0.373)	0.248 (0.169–0.335)	0.216 (0.137–0.308)	0.079 (0.074–0.084)
	60–69	0.399 (0.312–0.486)	0.348 (0.255–0.442)	0.325 (0.231–0.424)	0.170 (0.159–0.181)
	70–79	0.433 (0.269–0.587)	0.406 (0.231–0.574)	0.375 (0.192–0.558)	0.272 (0.243–0.301)
Female, by age group	15–39	0.138 (0.095–0.189)	0.138 (0.073–0.225)	0.125 (0.061–0.214)	0.018 (0.017–0.019)
	40–49	0.157 (0.115–0.205)	0.133 (0.092–0.182)	0.110 (0.071–0.159)	0.047 (0.044–0.050)
	50–59	0.168 (0.119–0.225)	0.178 (0.103–0.270)	0.157 (0.083–0.252)	0.062 (0.058–0.066)
	60–69	0.322 (0.224–0.423)	0.273 (0.173–0.383)	0.232 (0.131–0.349)	0.089 (0.083–0.095)
	70–79	0.162 (0.091–0.250)	0.123 (0.056–0.218)	N/D	0.125 (0.112–0.139)
By type of first cancer	Stomach	0.214 (0.158–0.275)	0.184 (0.129–0.246)	0.166 (0.111–0.230)	N/A
	Colorectal	0.200 (0.144–0.262)	0.170 (0.114–0.235)	0.152 (0.097–0.219)	N/A
	Liver	0.686 (0.384–0.863)	0.554 (0.199–0.805)	N/D	N/A
	Lung	0.309 (0.223–0.399)	0.241 (0.157–0.336)	0.157 (0.080–0.256)	N/A
	Thyroid	N/D	N/D	N/D	N/A
	Leukemia	0.218 (0.080–0.398)	0.114 (0.015–0.323)	N/D	N/A
	Prostate/testis	0.222 (0.139–0.317)	0.186 (0.104–0.286)	0.163 (0.082–0.266)	N/A
	Breast	0.178 (0.137–0.225)	0.150 (0.109–0.197)	0.124 (0.084–0.172)	N/A
	Uterus	0.112 (0.074–0.160)	0.137 (0.060–0.245)	0.125 (0.050–0.236)	N/A
	Ovary	0.306 (0.159–0.466)	0.237 (0.097–0.411)	0.189 (0.059–0.374)	N/A
	Others	0.344 (0.283–0.405)	0.309 (0.236–0.385)	0.277 (0.202–0.357)	N/A

*CIS* carcinoma in situ, *CI* confidence interval, *N/A* not applicable, *N/D* not determined due to an insufficient number of survivors with adequate follow-up time

### Visualizing the relationships between cancer types using Sankey diagrams

**Fig. 5:**
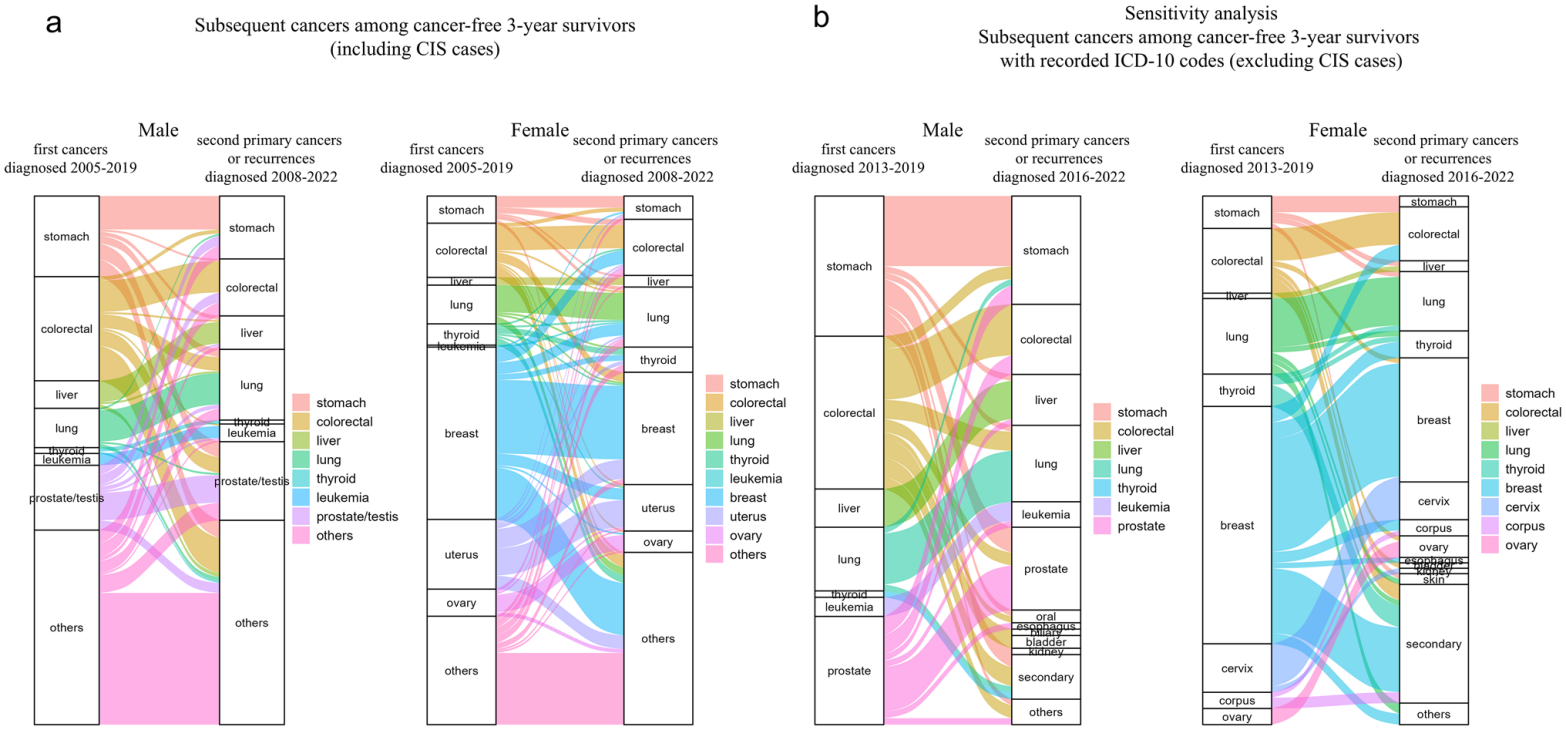
**a** Sankey diagrams showing the types of first cancers and second primary cancers or recurrences among “cancer-free 3-year survivors (including CIS cases)” **b** Sankey diagrams showing the types of first cancers and second primary cancers or recurrences among “cancer-free 3-year survivors (excluding CIS cases)” in the sensitivity analysis *CIS* carcinoma in situ

Finally, Sankey diagrams demonstrating the types of first cancers and second primary cancers or recurrences, as applicable, in “cancer-free 3-year survivors” are shown in Fig. 5. Most individuals whose first cancer types were “liver”, “lung”, “leukemia” or “ovary” received a second payment for the same cancer type. A sensitivity analysis on “cancer-free 3-year survivors” with recorded ICD-10 codes revealed that the second event was a relapse in all cases where the first cancer was liver cancer or leukemia, whereas it was a second primary cancer in more than half of the cases for prostate cancer.

## Discussion

In this study, we evaluated sex- and age group-specific RS and CRS, ASR by cancer type, as well as incidence of second primary cancers or recurrences, as applicable, among cancer survivors aged 15 to 79 years enrolled in private cancer insurance policies in Japan. With a median age at first cancer diagnosis of 59.0 years, the 5-year and 10-year ASR were 75.0% and 68.2%, respectively, and the 5-year conditional ASR among 5-year survivors was 90.1% for all cancers combined. When analyzed by type of first cancer, the 5-year ASR ranged from 34.1% for liver cancer to 96.5% for breast cancer. The cumulative incidence of second primary cancers or recurrences, as applicable, decreased as more time passed since the first cancer diagnosis; however, it remained high in “cancer-free 5-year survivors” among liver cancer survivors due to relapses.

The reported 5-year RS of individuals diagnosed with cancer between 2009–2011 was 62.0% for males and 66.9% for females, according to the population-based cancer registries in Japan [20]. The difference from our results may primarily be attributable to the age of the survivors. The national cancer registry indicates that over a quarter of the individuals newly diagnosed with cancer are aged ≥ 80 years [5], while only individuals under 80 years were enrolled in the cancer insurance policy in the current study. However, RS in this study may be higher than that in previous reports even when stratified by sex and age group: the 5-year net survival of individuals diagnosed in 2014–2015 was 80.7%, 73.4%, 69.0%, 66.6%, and 63.3% for males, and 86.9%, 87.3%, 81.2%, 74.6%, and 67.0% for females aged 15–39, 40–49, 50–59, 60–69, and 70–79 years, respectively, according to hospital-based registries in Japan [21]. Although the results are not directly comparable, the difference may be due to the better health profiles of the study population. Indeed, the cohort survival data of the entire insurance-enrolled population showed better survival than the national statistics from the same period (Supplementary Table 6 and Supplementary Fig. 2). Although we used an internal reference to minimize the effect of selection bias, such bias may have also influenced the characteristics of survivors for specific cancer types. For example, liver cancer survivors in this study may not represent typical cases, as individuals with hepatitis B or C infection, which were previously major causes of liver cancer in Japan [22], were ineligible. Additionally, participants in this study are likely to have higher socioeconomic status, higher health literacy, and to undergo cancer screening, leading to earlier cancer diagnoses. In contrast, a national survey reported that fewer than 50% of eligible people undergo cancer screening [5], despite national guidelines recommending screening for gastric, colorectal, lung, breast, and cervical cancer, which are generally provided by local governments in Japan [23, 24].

Notably, RS was higher in females than in males within the same age groups, a pattern that aligns with trends observed in previous reports [20, 21]. Previous studies suggested that worse survival in males can be partially attributed to their higher likelihood of engaging in high-risk behaviors such as smoking and their lower utilization of health care services compared to females [25]. Biological factors, including differences in sex hormones and immune function, may also play a role [26]. Further studies are warranted to elucidate the mechanisms underlying these sex-related disparities in cancer survival.

Although the risks of recurrence and of developing second primary cancers have been well described separately [8, 9, 27], the combined risk of second primary cancers or recurrences, as applicable, has rarely been addressed. The difference between the incidence rates of first cancers and second primary cancers or recurrences, as applicable, decreased with older age and increasing time since the first cancer. Notably, incidence rates remained high among liver cancer survivors, even after 5 cancer-free years, primarily due to recurrences, consistent with a previous study reporting a high risk of recurrence even after 5 recurrence-free years in liver cancer survivors [28]. As visualized in the Sankey diagram, the patterns of recurrences and second primary cancers varied by the type of first cancer, highlighting distinct risk profiles across cancer types.

The strengths of our study include the relatively long-term follow-up of cancer survivors. As these private cancer insurance policies offer benefit payments multiple times, cancer survivors are unlikely to terminate their contracts after being diagnosed with cancer, enabling long-term follow-up. Moreover, death is reliably recorded, unlike in some health insurance claims databases where such data may be incomplete. Furthermore, by comparing cancer survivors with over 400,000 individuals in the entire insurance-enrolled population during the same period, we were able to provide internally valid estimates of RS among survivors over time.

Several limitations should be noted. First, individuals aged ≥ 80 years could not enroll in the insurance policies, which likely led to an overestimation of the ASR, as RS is generally lower among survivors aged ≥ 80 years. Second, selection bias likely exists, as the study cohort comprises private cancer insurance policyholders who generally have better health profiles and higher socioeconomic status than the general population. Indeed, the cohort survival data from the entire insurance-enrolled population showed better survival than national statistics during the same period. Third, detailed information on cancer, including stage, pathological subtypes, and whether the second payment was for relapse or second primary cancer, was unavailable. Additionally, payment dates may not correspond precisely to the dates of diagnosis.

In conclusion, we estimated sex- and age group-specific RS, CRS, and ASR by cancer type, as well as incidence of second primary cancers or recurrences, as applicable, using a database of individuals enrolled in private cancer insurance policies. Although our findings may not be directly generalizable to the national cancer population, they offer valuable insights into long-term survival and subsequent cancer risks among privately insured populations, contributing to a broader understanding of cancer outcomes in Japan.

## Supplementary Information

Below is the link to the electronic supplementary material.

Supplementary file1 (PDF 1202 KB)

## Data Availability

The dataset used in this study is proprietary to Asahi Mutual Life Insurance Company and is not publicly available. Restrictions apply to the availability of these data, which were used under license for this study. Data are available from the corresponding author with the permission of Asahi Mutual Life Insurance Company.
