# Supplementary material for: Relative survival among cancer survivors enrolled in private cancer insurance in Japan, using the internal insurance-enrolled population as the reference
Source: Int J Clin Oncol. 2025 Sep 9;30(11):2178–91. doi: 10.1007/s10147-025-02871-6 (PMC12568848; doi:10.1007/s10147-025-02871-6)
Supplement: Supplementary file 1 — Supplementary file1 (PDF 1202 KB) [file 10147_2025_2871_MOESM1_ESM.pdf]

Supplementary Information

Supplementary Methods

Data sources and study population

Supplementary Table 2 provides a breakdown of ICD-10 codes for each disease category within the subgroup of cases with recorded ICD-10 codes. For example, the “colorectal cancer” code also includes a small number of cases classified as cancers of the small intestine, anus, or anal canal. Although the exact cancer site is unavailable for cases without recorded ICD-10 codes, we refer to all cancers classified under the “colorectal cancer” code as “colorectal cancer” in this study, since 97.8% of the cases with recorded ICD-10 codes were indeed colorectal cancer. These codes do not necessarily indicate the primary site of cancer; for example, liver metastases from colorectal cancer or invasion of colorectal cancer into neighboring organs were categorized as “others” rather than “colorectal”.

Outcome measures

The 5-year CRS was defined as the relative survival at x+5 years among “x-year survivors”.
The age-standardized relative survival (ASR) for each cancer type was estimated using population weights for five age groups (15–44, 45–54, 55–64, 65–74, and ≥75 years) defined by Corazziari *et al.* [1], although the ASR is likely overestimated due to the absence of participants aged ≥80 years. The International Cancer Survival Standards (ICSS) used for each cancer type are shown in Supplementary Table 1. For the ASR of all cancer sites combined, a single standard distribution defined by Corazziari *et al.* [1] was applied. The ASR could not be estimated for some cancer types due to an insufficient number of survivors in certain age groups with adequate follow-up time.
The date of the second payment was defined as the earliest payment occurring at least one year after the first payment. This approach was taken to distinguish cases in which individuals with multiple contracts might receive payments for the same diagnosis on different dates. For the analysis of second primary cancers or recurrences, as applicable, cancer survivors were followed from 3, 4, or 5 years after the first cancer diagnosis until the occurrence of a second payment, coverage termination, death, or March 31, 2022, whichever occurred first. For the analysis of first cancers for comparison, the “Entire insurance-enrolled population” was followed from the date of enrollment until the first cancer diagnosis, coverage termination, death, or March 31, 2022, whichever occurred first (Supplementary Fig. 1). For individuals with recorded ICD-10 codes for the first cancer, a second payment with the same ICD-10 category as the first cancer, as defined in Supplementary Table 1, or an ICD-10 code indicating a secondary cancer, was considered a relapse of the first cancer.

Cohort survival data

Survival rates by age were estimated for the entire insurance-enrolled population who enrolled between 2005 and 2011, using the *survfit()* function from the *survival* package in R. For comparison, national statistics for corresponding ages in 2005 and 2011 were obtained from the cohort survival tables published by the National Cancer Center Japan [2].

Sensitivity analyses

Two sensitivity analyses were performed to estimate relative survival (RS). First, we excluded carcinoma in situ (CIS) cases by restricting the analysis to a subgroup of individuals with available ICD-10 codes, which were recorded from 2013 onward. Accordingly, the survival rates of the entire insurance-enrolled population enrolled in or after 2013 were used as the internal reference. Second, to better approximate net survival and account for potential overestimation due to the inclusion of cancer cases in the Ederer II-based reference, we performed an analysis in which individuals in the internal reference population were censored at the time of cancer diagnosis. For this analysis, individuals in the entire insurance-enrolled population were followed from the date of enrollment until the first cancer diagnosis, death, coverage termination, or March 31, 2022, whichever occurred first. This censoring strategy was also applied to the subgroup with recorded ICD-10 codes, excluding CIS cases.

Cohort and period analyses

To obtain more up-to-date estimates of survival, period analyses [3] were conducted. Specifically, period analyses were performed for individuals who were followed up between 2012–2016 and 2017–2021, respectively, along with conventional cohort analyses for those diagnosed between 2005–2011 and 2012–2017. For each analysis, survival rates of the “Entire insurance-enrolled population” during the same periods were used to estimate RS. Illustrations of the time scales in cohort and period analyses are shown in Supplementary Fig. 5a.

Statistical analysis

Background characteristics of the participants were summarized using frequencies and proportions for categorical variables, and medians and interquartile ranges (IQRs) for continuous variables. Incidence rates of second primary cancers or recurrences, as applicable, per 1,000 person-years were calculated using the *epi.conf()* function from the *epiR* package in R. The frequencies of cancer types for the first and second payments were visualized using Sankey diagrams created with the *ggsankey* package in R.

References

1. Corazziari I, Quinn M, Capocaccia R (2004) Standard cancer patient population for age standardising survival ratios. Eur J Cancer 40 (15):2307-2316. https://doi.org/10.1016/j.ejca.2004.07.002
2. Cohort survival table. Cancer Information Service, National Cancer Center, Japan. https://ganjoho.jp/reg_stat/statistics/data/cohort/index.html. Accessed 24 June 2025
3. Brenner H, Gefeller O (1997) Deriving more up-to-date estimates of long-term patient survival. J Clin Epidemiol 50 (2):211-216. https://doi.org/10.1016/s0895-4356(97)00280-1

Supplementary Table 1 Corresponding ICD-10 codes used in the main analysis (including CIS cases) and in the sensitivity analysis (excluding CIS cases)

All survivors (including CIS cases)			All survivors with recorded ICD-10 codes for sensitivity analyses (excluding CIS cases)		
Category name of cancer type in this study	Corresponding ICD-10 codes	ICSS population weights used for calculating age-standardized relative survival	Category name of cancer type in this study	ICD-10 codes	ICSS population weights used for calculating age-standardized relative survival
all	C00-C97, D00-D09	single standard [a]	all	C00-C97	single standard [a]
stomach	Stomach (C16, D002)	ICSS 1	stomach	C16	ICSS 1
colorectal	Small intestine (C17), Colon and rectum (C18-C20, D010-D012), Anus and anal canal (C21, D013), Other unspecified parts of intestine (D014)	ICSS 1	colorectal	C18-C20	ICSS 1
liver	Liver (C22)	ICSS 1	liver	C22	ICSS 1
lung	Lung (C33, C34)	ICSS 1	lung	C33, C34	ICSS 1
breast	Breast (C50, D05)	ICSS 1	breast	C50	ICSS 1
uterus	Cervix uteri (C53, D06), Corpus uteri (C54, D070), Uterus (C55, D073), Placenta (C58)	ICSS 2	cervix uteri	C53	ICSS 2
			corpus uteri	C54	ICSS 1
leukemia	Leukemia (C91-C95)	ICSS 1	leukemia	C91-C95	ICSS 1
ovary	Vulva (C51, D071), Vagina (C52, D072), Ovary (C56), Other and unspecified female genital organs (C57)	ICSS 1	ovary	C56	ICSS 1
prostate/testis	Penis (C60, D074), Prostate (C61, D075), Testis (C62), Other and unspecified male genital organs (C63)	ICSS 1	prostate	C61	ICSS 1
			testis	C62	ICSS 3
thyroid	Thyroid (C73)	ICSS 2	thyroid	C73	ICSS 2
others	ICD10 codes starting C- (including secondary cancer) and D0- except for those listed above	ICSS 1	oral (lip, oral cavity and pharynx)	C00-C14	ICSS 1
			esophagus	C15	ICSS 1
			biliary	C23, C24	ICSS 1
			pancreas	C25	ICSS 1
			bladder	C67	ICSS 1
			kidney (urinary organs excluding bladder)	C64-C66, C68	ICSS 1
			lymphoma	C81-C85, C96	ICSS 1
			myeloma	C88-C90	ICSS 1
			brain (central nervous system)	C70-C72	ICSS 2
			skin	C43, C44	ICSS 1
			secondary cancer	C77-C79	ICSS 1

ICD-10, International Classification of Diseases, Tenth Revision; CIS, carcinoma in situ; ICSS, International Cancer Survival Standard

[a] single standard distribution defined by Corazziari et al [1]

Supplementary Table 2 Percentages of ICD-10 codes among cancer survivors with recorded ICD-10 codes by cancer type category in the main analysis (including CIS cases, diagnosed 2013-2022)

Category name of cancer type in this study	ICD10 code	Site of cancer	All	Age group, 15-39	Age group, 40-49	Age group, 50-59	Age group, 60-69	Age group, 70-79
stomach	C16	Stomach	100.0	100.0	100.0	100.0	100.0	100.0
colorectal	C18-C20	Colorectal	93.0	97.9	91.1	91.9	94.5	92.3
	D010-D012	Colorectal (in situ)	4.8	0.0	5.6	5.5	4.1	5.2
	C17	Small intestine	1.4	0.0	2.8	1.2	0.9	1.7
	C21	Anus and anal canal	0.6	2.1	0.0	1.2	0.5	0.3
	D013	Anus and anal canal (in situ)	0.2	0.0	0.6	0.3	0.0	0.0
	D014	Other and unspecified parts of intestine (in situ)	0.1	0.0	0.0	0.0	0.0	0.3
liver	C22	Liver	100.0	100.0	100.0	100.0	100.0	100.0
lung	C33, C34	Lung	100.0	100.0	100.0	100.0	100.0	100.0
thyroid	C73	Thyroid	100.0	100.0	100.0	100.0	100.0	100.0
leukemia	C91-C95	Leukemia	100.0	100.0	100.0	100.0	100.0	100.0
prostate/testis	C61	Prostate	90.5	0.0	14.3	91.7	98.8	100.0
	C62	Testis	9.5	100.0	85.7	8.3	1.2	0.0
breast	C50	Breast	94.7	90.9	96.9	92.8	94.7	95.9
	D05	Breast (in situ)	5.3	9.1	3.1	7.2	5.3	4.1
uterus	C53	Cervix uteri	21.7	13.9	22.9	28.1	40.3	27.3
	D06	Cervix uteri (in situ)	56.2	80.2	55.9	27.2	16.1	9.1
	C54	Corpus uteri	20.6	4.5	19.5	43.9	41.9	63.6
	C55	Uterus, part unspecified	0.3	0.3	0.0	0.0	1.6	0.0
	D070, D073	Corpus uteri (in situ), uterus (in situ)	1.0	0.7	1.7	0.9	0.0	0.0
	C58	Placenta	0.1	0.3	0.0	0.0	0.0	0.0
ovary	C56	Ovary	94.2	100.0	100.0	96.3	90.5	78.6
	C57	Other and unspecified female genital organs	2.9	0.0	0.0	3.7	4.8	7.1
	C51	Vulva	1.9	0.0	0.0	0.0	4.8	7.1
	D071	Vulva (in situ)	1.0	0.0	0.0	0.0	0.0	7.1
others	C00-C14	Oral (lip, oral cavity and pharynx)	8.9	18.0	12.6	9.3	7.6	6.6
	C15	Esophagus	8.5	1.0	6.9	10.8	10.2	7.0
	C23, C24	Biliary	4.1	0.0	1.7	2.6	4.1	7.3
	C25	Pancreas	10.9	3.0	8.6	9.9	12.1	12.9
	C43, C44	Skin	8.0	10.0	10.3	6.1	7.0	9.2
	C64-C66, C68	Kidney (urinary organs excluding bladder)	11.7	10.0	11.5	14.9	10.7	10.8
	C67	Bladder	8.5	5.0	5.7	10.2	9.4	8.0
	C70-C72	Brain (central nervous system)	2.5	14.0	3.4	2.0	1.0	1.6
	C77-C79	Secondary cancer	9.3	1.0	4.0	9.9	11.3	10.6
	C81-C85, C96	Lymphoma	12.0	21.0	14.9	9.9	10.7	11.7
	C88-C90	Myeloma	2.6	1.0	3.4	2.9	1.6	3.8
	D00-D09	Carcinoma in situ	3.5	3.0	2.3	2.3	4.5	4.0

CIS, carcinoma in situ; ICD-10, International Classification of Diseases, Tenth Revision

Supplementary Table 3 Characteristics of "All survivors (diagnosed 2005-2022, including CIS cases)" stratified by type of first cancer

Type of first cancer		Total	stomach	colorectal	liver	lung	thyroid	leukemia	prostate/testis	breast	uterus	ovary	others
n		8846	813	1655	172	669	215	119	550	1447	1010	145	2051
Age, median [IQR]		59.0 [48.0, 67.0]	63.0 [54.0, 70.0]	60.0 [52.0, 68.0]	61.0 [55.0, 69.0]	64.0 [57.0, 70.0]	49.0 [39.0, 58.0]	56.0 [41.5, 65.0]	66.0 [60.0, 72.0]	53.0 [46.0, 62.0]	42.0 [35.0, 51.0]	54.0 [43.0, 63.0]	61.0 [51.0, 68.0]
Age group, n(%)	15-39	1017 (11.5)	41 (5.0)	72 (4.4)	7 (4.1)	15 (2.2)	57 (26.5)	26 (21.8)	42 (7.6)	118 (8.2)	433 (42.9)	23 (15.9)	183 (8.9)
	40-49	1524 (17.2)	85 (10.5)	235 (14.2)	15 (8.7)	49 (7.3)	56 (26.0)	17 (14.3)	17 (3.1)	447 (30.9)	306 (30.3)	36 (24.8)	261 (12.7)
	50-59	2123 (24.0)	188 (23.1)	477 (28.8)	51 (29.7)	151 (22.6)	54 (25.1)	25 (21.0)	76 (13.8)	421 (29.1)	156 (15.4)	41 (28.3)	483 (23.5)
	60-69	2526 (28.6)	291 (35.8)	550 (33.2)	59 (34.3)	264 (39.5)	38 (17.7)	34 (28.6)	218 (39.6)	300 (20.7)	86 (8.5)	28 (19.3)	658 (32.1)
	70-79	1656 (18.7)	208 (25.6)	321 (19.4)	40 (23.3)	190 (28.4)	10 (4.7)	17 (14.3)	197 (35.8)	161 (11.1)	29 (2.9)	17 (11.7)	466 (22.7)
Sex, n(%)	female	4934 (55.8)	313 (38.5)	731 (44.2)	51 (29.7)	250 (37.4)	153 (71.2)	54 (45.4)	0 (0.0)	1439 (99.4)	1010 (100.0)	145 (100.0)	788 (38.4)
Year of first cancer, n(%)	2005-2011	1522 (17.2)	180 (22.1)	249 (15.0)	39 (22.7)	113 (16.9)	12 (5.6)	24 (20.2)	74 (13.5)	232 (16.0)	195 (19.3)	33 (22.8)	371 (18.1)
	2012-2017	3427 (38.7)	337 (41.5)	652 (39.4)	64 (37.2)	261 (39.0)	100 (46.5)	47 (39.5)	203 (36.9)	610 (42.2)	369 (36.5)	51 (35.2)	733 (35.7)
	2018-2022	3897 (44.1)	296 (36.4)	754 (45.6)	69 (40.1)	295 (44.1)	103 (47.9)	48 (40.3)	273 (49.6)	605 (41.8)	446 (44.2)	61 (42.1)	947 (46.2)
Second payment, n(%)		1159 (13.1)	106 (13.0)	205 (12.4)	47 (27.3)	115 (17.2)	18 (8.4)	20 (16.8)	55 (10.0)	150 (10.4)	69 (6.8)	29 (20.0)	345 (16.8)
Death before second payment, n(%)		1246 (14.1)	160 (19.7)	136 (8.2)	77 (44.8)	219 (32.7)	2 (0.9)	30 (25.2)	24 (4.4)	48 (3.3)	40 (4.0)	19 (13.1)	491 (23.9)
Overall death, n(%)		1772 (20.0)	210 (25.8)	228 (13.8)	103 (59.9)	289 (43.2)	2 (0.9)	41 (34.5)	39 (7.1)	99 (6.8)	67 (6.6)	39 (26.9)	655 (31.9)
Follow-up years, median (IQR)		3.40 [1.34, 6.76]	3.70 [1.19, 7.71]	3.72 [1.75, 6.89]	1.34 [0.19, 4.02]	1.92 [0.61, 4.16]	4.23 [2.20, 6.97]	2.44 [1.27, 6.34]	3.47 [1.77, 6.65]	4.50 [2.22, 7.67]	4.12 [2.09, 8.27]	3.22 [1.59, 6.25]	2.47 [0.84, 5.95]

CIS, carcinoma in situ; IQR, interquartile range

Supplementary Table 4 Characteristics of "All survivors with recorded ICD-10 codes (diagnosed 2013-2022, excluding CIS cases)", stratified by sex and age group

		**All**	**Male**						**Female**					
**Age group**		**Total**	**Total**	**15-39**	**40-49**	**50-59**	**60-69**	**70-79**	**Total**	**15-39**	**40-49**	**50-59**	**60-69**	**70-79**
n		6110	2880	141	334	661	1007	737	3230	275	599	767	905	684
Age, median [IQR]		61.0 [51.0, 69.0]	63.0 [54.0, 70.0]	35.0 [30.0, 37.0]	46.0 [43.0, 48.0]	55.0 [53.0, 58.0]	65.0 [62.5, 67.0]	73.0 [71.0, 76.0]	59.0 [49.0, 68.0]	35.0 [31.0, 37.0]	45.0 [43.0, 47.0]	55.0 [52.0, 57.0]	64.0 [62.0, 67.0]	73.0 [71.0, 76.0]
Sex, n(%)	female	3230 (52.9)	-	-	-	-	-	-	3230 (100.0)	275 (100.0)	599 (100.0)	767 (100.0)	905 (100.0)	684 (100.0)
Type of first cancer, n(%)	stomach	568 (9.3)	347 (12.0)	12 (8.5)	27 (8.1)	72 (10.9)	140 (13.9)	96 (13.0)	221 (6.8)	13 (4.7)	15 (2.5)	41 (5.3)	77 (8.5)	75 (11.0)
	colorectal	1205 (19.7)	660 (22.9)	29 (20.6)	110 (32.9)	186 (28.1)	219 (21.7)	116 (15.7)	545 (16.9)	18 (6.5)	53 (8.8)	130 (16.9)	196 (21.7)	148 (21.6)
	liver	121 (2.0)	81 (2.8)	1 (0.7)	8 (2.4)	28 (4.2)	30 (3.0)	14 (1.9)	40 (1.2)	1 (0.4)	2 (0.3)	10 (1.3)	9 (1.0)	18 (2.6)
	lung	520 (8.5)	309 (10.7)	2 (1.4)	23 (6.9)	62 (9.4)	132 (13.1)	90 (12.2)	211 (6.5)	7 (2.5)	10 (1.7)	40 (5.2)	75 (8.3)	79 (11.5)
	thyroid	185 (3.0)	58 (2.0)	11 (7.8)	20 (6.0)	12 (1.8)	11 (1.1)	4 (0.5)	127 (3.9)	36 (13.1)	28 (4.7)	35 (4.6)	23 (2.5)	5 (0.7)
	leukemia	82 (1.3)	44 (1.5)	6 (4.3)	7 (2.1)	10 (1.5)	13 (1.3)	8 (1.1)	38 (1.2)	7 (2.5)	5 (0.8)	5 (0.7)	15 (1.7)	6 (0.9)
	prostate	399 (6.5)	399 (13.9)	0 (0.0)	2 (0.6)	55 (8.3)	169 (16.8)	173 (23.5)	N/A	N/A	N/A	N/A	N/A	N/A
	breast	1051 (17.2)	6 (0.2)	0 (0.0)	0 (0.0)	1 (0.2)	2 (0.2)	3 (0.4)	1045 (32.4)	80 (29.1)	312 (52.1)	284 (37.0)	231 (25.5)	138 (20.2)
	cervix uteri	157 (2.6)	N/A	N/A	N/A	N/A	N/A	N/A	157 (4.9)	40 (14.5)	54 (9.0)	32 (4.2)	25 (2.8)	6 (0.9)
	corpus uteri	149 (2.4)	N/A	N/A	N/A	N/A	N/A	N/A	149 (4.6)	13 (4.7)	46 (7.7)	50 (6.5)	26 (2.9)	14 (2.0)
	ovary	97 (1.6)	N/A	N/A	N/A	N/A	N/A	N/A	97 (3.0)	17 (6.2)	24 (4.0)	26 (3.4)	19 (2.1)	11 (1.6)
	oral	139 (2.3)	96 (3.3)	9 (6.4)	15 (4.5)	26 (3.9)	28 (2.8)	18 (2.4)	43 (1.3)	9 (3.3)	7 (1.2)	6 (0.8)	11 (1.2)	10 (1.5)
	esophagus	132 (2.2)	106 (3.7)	1 (0.7)	9 (2.7)	30 (4.5)	43 (4.3)	23 (3.1)	26 (0.8)	0 (0.0)	3 (0.5)	7 (0.9)	9 (1.0)	7 (1.0)
	biliary	64 (1.0)	39 (1.4)	0 (0.0)	2 (0.6)	5 (0.8)	14 (1.4)	18 (2.4)	25 (0.8)	0 (0.0)	1 (0.2)	4 (0.5)	7 (0.8)	13 (1.9)
	pancreas	169 (2.8)	88 (3.1)	1 (0.7)	8 (2.4)	20 (3.0)	33 (3.3)	26 (3.5)	81 (2.5)	2 (0.7)	7 (1.2)	14 (1.8)	29 (3.2)	29 (4.2)
	bladder	132 (2.2)	104 (3.6)	5 (3.5)	8 (2.4)	32 (4.8)	39 (3.9)	20 (2.7)	28 (0.9)	0 (0.0)	2 (0.3)	3 (0.4)	9 (1.0)	14 (2.0)
	kidney	182 (3.0)	118 (4.1)	6 (4.3)	16 (4.8)	35 (5.3)	32 (3.2)	29 (3.9)	64 (2.0)	4 (1.5)	4 (0.7)	16 (2.1)	23 (2.5)	17 (2.5)
	lymphoma	186 (3.0)	96 (3.3)	14 (9.9)	19 (5.7)	21 (3.2)	21 (2.1)	21 (2.8)	90 (2.8)	7 (2.5)	7 (1.2)	13 (1.7)	34 (3.8)	29 (4.2)
	myeloma	41 (0.7)	24 (0.8)	0 (0.0)	5 (1.5)	8 (1.2)	4 (0.4)	7 (0.9)	17 (0.5)	1 (0.4)	1 (0.2)	2 (0.3)	4 (0.4)	9 (1.3)
	brain	39 (0.6)	30 (1.0)	11 (7.8)	5 (1.5)	5 (0.8)	3 (0.3)	6 (0.8)	9 (0.3)	3 (1.1)	1 (0.2)	2 (0.3)	2 (0.2)	1 (0.1)
	testis	42 (0.7)	42 (1.5)	23 (16.3)	12 (3.6)	5 (0.8)	2 (0.2)	0 (0.0)	N/A	N/A	N/A	N/A	N/A	N/A
	skin	124 (2.0)	71 (2.5)	4 (2.8)	16 (4.8)	12 (1.8)	18 (1.8)	21 (2.8)	53 (1.6)	6 (2.2)	2 (0.3)	9 (1.2)	18 (2.0)	18 (2.6)
	secondary	145 (2.4)	65 (2.3)	0 (0.0)	2 (0.6)	16 (2.4)	21 (2.1)	26 (3.5)	80 (2.5)	1 (0.4)	5 (0.8)	18 (2.3)	37 (4.1)	19 (2.8)
	others	181 (3.0)	97 (3.4)	6 (4.3)	20 (6.0)	20 (3.0)	33 (3.3)	18 (2.4)	84 (2.6)	10 (3.6)	10 (1.7)	20 (2.6)	26 (2.9)	18 (2.6)
Year of first cancer, n(%)	2013-2017	2654 (43.4)	1227 (42.6)	79 (56.0)	157 (47.0)	300 (45.4)	458 (45.5)	233 (31.6)	1427 (44.2)	157 (57.1)	310 (51.8)	353 (46.0)	385 (42.5)	222 (32.5)
	2018-2022	3456 (56.6)	1653 (57.4)	62 (44.0)	177 (53.0)	361 (54.6)	549 (54.5)	504 (68.4)	1803 (55.8)	118 (42.9)	289 (48.2)	414 (54.0)	520 (57.5)	462 (67.5)
Second payment, n(%)		673 (11.0)	335 (11.6)	19 (13.5)	35 (10.5)	77 (11.6)	130 (12.9)	74 (10.0)	338 (10.5)	36 (13.1)	58 (9.7)	71 (9.3)	110 (12.2)	63 (9.2)
Death before second payment, n(%)		881 (14.4)	514 (17.8)	13 (9.2)	41 (12.3)	110 (16.6)	185 (18.4)	165 (22.4)	367 (11.4)	14 (5.1)	28 (4.7)	80 (10.4)	129 (14.3)	116 (17.0)
Overall death, n(%)		1169 (19.1)	655 (22.7)	20 (14.2)	54 (16.2)	143 (21.6)	240 (23.8)	198 (26.9)	514 (15.9)	22 (8.0)	53 (8.8)	110 (14.3)	181 (20.0)	148 (21.6)
Follow-up years, median (IQR)		2.82 [1.14, 4.99]	2.57 [0.96, 4.76]	3.90 [1.96, 6.17]	3.20 [1.44, 5.51]	2.74 [1.00, 5.19]	2.55 [0.93, 4.71]	2.10 [0.74, 3.86]	3.01 [1.34, 5.17]	4.26 [2.06, 6.29]	3.92 [2.01, 6.04]	3.18 [1.34, 5.61]	2.89 [1.26, 4.85]	2.20 [0.84, 3.86]

ICD-10, International Classification of Diseases, Tenth Revision; CIS, carcinoma in situ; IQR, interquartile range ; N/A, not applicable

Supplementary Table 5 Characteristics of "All survivors (diagnosed 2005-2022, including CIS cases)" with or without death

Variable	Group	Total				Male				Female			
		Total	Death		P value	Total	Death		P value	Total	Death		P value
			no	yes			no	yes			no	yes	
n		8846	7074	1772		3912	2893	1019		4934	4181	753	
Age, median [IQR]		59.0 [48.0, 67.0]	57.0 [46.0, 66.0]	63.0 [54.0, 69.0]	<0.001	61.5 [53.0, 68.0]	61.0 [52.0, 68.0]	63.0 [55.0, 69.0]	<0.001	55.0 [44.0, 66.0]	53.0 [43.0, 64.0]	62.0 [53.0, 69.0]	<0.001
Age group , n(%)	15-39	1017 (11.5)	930 (13.1)	87 (4.9)	<0.001	246 (6.3)	201 (6.9)	45 (4.4)	<0.001	771 (15.6)	729 (17.4)	42 (5.6)	<0.001
	40-49	1524 (17.2)	1323 (18.7)	201 (11.3)		481 (12.3)	382 (13.2)	99 (9.7)		1043 (21.1)	941 (22.5)	102 (13.5)	
	50-59	2123 (24.0)	1702 (24.1)	421 (23.8)		973 (24.9)	724 (25.0)	249 (24.4)		1150 (23.3)	978 (23.4)	172 (22.8)	
	60-69	2526 (28.6)	1901 (26.9)	625 (35.3)		1353 (34.6)	980 (33.9)	373 (36.6)		1173 (23.8)	921 (22.0)	252 (33.5)	
	70-79	1656 (18.7)	1218 (17.2)	438 (24.7)		859 (22.0)	606 (21.0)	253 (24.8)		797 (16.2)	612 (14.6)	185 (24.6)	
Sex, n(%)	female	4934 (55.8)	4181 (59.1)	753 (42.5)	<0.001	N/A	N/A	N/A	N/A	4934 (100.0)	4181 (100.0)	753 (100.0)	N/A
Type of first cancer, n(%)	stomach	813 (9.2)	603 (8.5)	210 (11.9)	<0.001	500 (12.8)	374 (12.9)	126 (12.4)	<0.001	313 (6.3)	229 (5.5)	84 (11.2)	<0.001
	colorectal	1655 (18.7)	1427 (20.2)	228 (12.9)		924 (23.6)	785 (27.2)	139 (13.6)		731 (14.8)	642 (15.4)	89 (11.8)	
	liver	172 (1.9)	69 (1.0)	103 (5.8)		121 (3.1)	50 (1.7)	71 (7.0)		51 (1.0)	19 (0.5)	32 (4.2)	
	lung	669 (7.6)	380 (5.4)	289 (16.3)		419 (10.7)	201 (7.0)	218 (21.4)		250 (5.1)	179 (4.3)	71 (9.4)	
	thyroid	215 (2.4)	213 (3.0)	2 (0.1)		62 (1.6)	62 (2.1)	0 (0.0)		153 (3.1)	151 (3.6)	2 (0.3)	
	leukemia	119 (1.3)	78 (1.1)	41 (2.3)		65 (1.7)	43 (1.5)	22 (2.2)		54 (1.1)	35 (0.8)	19 (2.5)	
	prostate/testis	550 (6.2)	511 (7.2)	39 (2.2)		550 (14.1)	511 (17.7)	39 (3.8)		N/A	N/A	N/A	
	breast	1447 (16.4)	1348 (19.1)	99 (5.6)		8 (0.2)	7 (0.2)	1 (0.1)		1439 (29.2)	1341 (32.1)	98 (13.0)	
	uterus	1010 (11.4)	943 (13.3)	67 (3.8)		N/A	N/A	N/A		1010 (20.5)	943 (22.6)	67 (8.9)	
	ovary	145 (1.6)	106 (1.5)	39 (2.2)		N/A	N/A	N/A		145 (2.9)	106 (2.5)	39 (5.2)	
	others	2051 (23.2)	1396 (19.7)	655 (37.0)		1263 (32.3)	860 (29.7)	403 (39.5)		788 (16.0)	536 (12.8)	252 (33.5)	
Year of first cancer diagnosis, n(%)	2005-2011	1522 (17.2)	1076 (15.2)	446 (25.2)	<0.001	688 (17.6)	414 (14.3)	274 (26.9)	<0.001	834 (16.9)	662 (15.8)	172 (22.8)	<0.001
	2012-2017	3427 (38.7)	2627 (37.1)	800 (45.1)		1498 (38.3)	1067 (36.9)	431 (42.3)		1929 (39.1)	1560 (37.3)	369 (49.0)	
	2018-2022	3897 (44.1)	3371 (47.7)	526 (29.7)		1726 (44.1)	1412 (48.8)	314 (30.8)		2171 (44.0)	1959 (46.9)	212 (28.2)	
Second payment, n(%)	Yes	1159 (13.1)	633 (8.9)	526 (29.7)	<0.001	579 (14.8)	316 (10.9)	263 (25.8)	<0.001	580 (11.8)	317 (7.6)	263 (34.9)	<0.001
Follow-up years, median [IQR]		3.40 [1.34, 6.76]	4.18 [2.03, 7.59]	1.07 [0.29, 2.56]	<0.001	3.02 [1.04, 6.14]	4.02 [1.84, 7.21]	0.97 [0.21, 2.27]	<0.001	3.76 [1.67, 7.20]	4.32 [2.15, 7.77]	1.22 [0.39, 2.74]	<0.001

CIS, carcinoma in situ; IQR, interquartile range; N/A, not applicable

Supplementary Table 6 Cohort survival data of the entire insurance-enrolled population (year 2005-2011) and the corresponding national statistics

	Male					
	Survival at 5 years (95%CI)			Survival at 10 years (95% CI)		
Age	Study population (year 2005-2011)	National statistics* (year 2005)	National statistics* (year 2011)	Study population (year 2005-2011)	National statistics* (year 2005)	National statistics* (year 2011)
15	0.998 (0.994–1.000)	0.998	0.999	0.996 (0.991–1.000)	0.995	0.996
20	0.997 (0.995–1.000)	0.997	0.997	0.995 (0.992–0.998)	0.994	0.995
25	0.998 (0.996–0.999)	0.997	0.997	0.995 (0.992–0.998)	0.993	0.994
30	0.997 (0.995–0.999)	0.996	0.997	0.993 (0.990–0.997)	0.992	0.993
35	1.000 (0.999–1.000)	0.995	0.996	0.998 (0.996–1.000)	0.987	0.990
40	0.995 (0.991–0.998)	0.992	0.993	0.989 (0.984–0.994)	0.981	0.984
45	0.992 (0.988–0.997)	0.987	0.989	0.986 (0.979–0.992)	0.969	0.974
50	0.987 (0.981–0.993)	0.980	0.983	0.972 (0.962–0.981)	0.952	0.959
55	0.986 (0.978–0.993)	0.967	0.973	0.966 (0.953–0.979)	0.923	0.935
60	0.974 (0.962–0.986)	0.951	0.956	0.936 (0.916–0.957)	0.885	0.895
65	0.973 (0.957–0.989)	0.928	0.932	0.909 (0.880–0.940)	0.832	0.840
70	0.968 (0.944–0.994)	0.884	0.898	0.892 (0.846–0.941)	0.730	0.760
	Female					
	Survival at 5 years (95%CI)			Survival at 10 years (95%CI)		
Age	Study population (year 2005-2011)	National statistics* (year 2005)	National statistics* (year 2011)	Study population (year 2005-2011)	National statistics* (year 2005)	National statistics* (year 2011)
15	1.000 (1.000–1.000)	0.999	0.999	1.000 (1.000–1.000)	0.998	0.998
20	1.000 (1.000–1.000)	0.999	0.999	1.000 (1.000–1.000)	0.997	0.998
25	0.999 (0.997–1.000)	0.998	0.998	0.998 (0.996–1.000)	0.996	0.997
30	0.998 (0.997–1.000)	0.998	0.998	0.998 (0.997–1.000)	0.995	0.996
35	0.997 (0.995–1.000)	0.997	0.997	0.997 (0.994–0.999)	0.993	0.994
40	0.997 (0.994–1.000)	0.996	0.996	0.991 (0.985–0.996)	0.989	0.990
45	0.996 (0.992–0.999)	0.993	0.994	0.990 (0.985–0.995)	0.984	0.985
50	0.996 (0.992–0.999)	0.990	0.991	0.990 (0.985–0.996)	0.977	0.979
55	0.993 (0.988–0.998)	0.986	0.987	0.982 (0.973–0.990)	0.966	0.970
60	0.993 (0.988–0.999)	0.980	0.981	0.971 (0.959–0.983)	0.952	0.955
65	0.989 (0.982–0.997)	0.970	0.972	0.953 (0.936–0.970)	0.925	0.931
70	0.992 (0.982–1.000)	0.948	0.955	0.936 (0.909–0.963)	0.869	0.885

*National statistics are based on data available at the Center for Cancer Control and Information Services, National Cancer Center, Japan (https://ganjoho.jp/reg_stat/statistics/data/cohort/index.html).

CI, confidence interval

Supplementary Table 7 Sex- and age group-specific 5-year and 10-year relative survival and 5-year conditional relative survival among cancer survivors in the main analyses (including CIS cases) and sensitivity analyses (excluding CIS cases), with censoring at first cancer diagnosis in the reference population

	Age group	Relative survival (95%CI)		5-year conditional relative survival (95%CI)				
		5-year RS	10-year RS	1-year survivors	2-year survivors	3-year survivors	4-year survivors	5-year survivors
Main analysis (diagnosed 2005-2022, including CIS cases)								
Male, by age group	15-39	81.8 (76.7-87.1)	78.8 (73.2-84.9)	88.9 (84.4-93.7)	92.1 (87.8-96.7)	92.1 (87.5-96.9)	95.0 (91.0-99.3)	96.4 (92.7-100.2)
	40-49	77.3 (73.1-81.6)	76.1 (71.6-80.8)	86.7 (82.9-90.6)	90.5 (86.7-94.5)	95.9 (92.8-99.1)	96.6 (93.7-99.7)	98.5 (96.1-100.6)
	50-59	73.0 (69.8-76.3)	67.3 (63.2-71.7)	81.0 (77.7-84.4)	87.2 (84.0-90.5)	91.0 (88.1-94.1)	92.5 (89.0-96.0)	92.3 (88.3-96.4)
	60-69	70.4 (67.4-73.5)	64.4 (60.0-69.2)	80.6 (77.4-83.9)	86.3 (83.1-89.7)	89.1 (85.6-92.6)	92.6 (88.9-96.4)	91.5 (86.5-96.8)
	70-79	66.1 (61.4-71.2)	55.2 (45.6-66.7)	78.0 (72.2-84.2)	78.9 (71.5-87.1)	78.9 (69.9-89.1)	82.9 (73.6-93.4)	83.5 (70.1-99.4)
Female, by age group	15-39	94.8 (93.0-96.6)	93.0 (90.7-95.4)	96.3 (94.8-97.9)	97.5 (96.0-99.0)	98.0 (96.5-99.5)	97.7 (95.9-99.5)	98.2 (96.5-99.9)
	40-49	89.9 (87.8-92.0)	86.5 (83.7-89.4)	91.0 (88.8-93.2)	92.3 (90.1-94.6)	94.7 (92.6-96.8)	95.7 (93.6-97.9)	96.3 (94.1-98.5)
	50-59	84.5 (82.1-87.0)	80.3 (77.2-83.6)	90.0 (87.7-92.4)	91.6 (89.3-94.0)	93.7 (91.4-96.0)	94.7 (92.4-97.0)	95.0 (92.4-97.6)
	60-69	76.5 (73.6-79.5)	70.3 (65.9-74.9)	83.4 (80.4-86.4)	89.1 (86.1-92.1)	92.0 (89.1-95.1)	92.6 (89.2-96.1)	91.9 (87.3-96.7)
	70-79	70.9 (66.7-75.5)	65.4 (57.6-74.2)	79.2 (74.2-84.4)	84.6 (79.6-90.0)	89.6 (84.0-95.5)	91.4 (84.4-99.0)	92.1 (82.5-102.9)
Sensitivity analysis (individuals with recorded ICD-10 codes, diagnosed 2013-2022, excluding CIS cases)								
Male with ICD-10 codes, by age group	15-39	84.5 (77.9-91.7)	N/D	89.2 (82.3-96.7)	88.5 (79.9-97.9)	88.5 (79.9-98.0)	N/D	N/D
	40-49	82.5 (77.9-87.4)	N/D	89.9 (85.8-94.2)	90.8 (85.0-97.1)	94.9 (89.4-100.7)	N/D	N/D
	50-59	74.4 (70.3-78.7)	N/D	83.1 (78.8-87.6)	88.7 (84.5-93.2)	91.1 (87.1-95.4)	N/D	N/D
	60-69	71.4 (67.7-75.2)	N/D	82.5 (78.6-86.6)	88.9 (85.0-93.0)	92.0 (87.3-96.9)	N/D	N/D
	70-79	66.6 (61.4-72.4)	N/D	78.6 (71.9-85.8)	85.1 (77.0-94.1)	86.9 (69.8-108.0)	N/D	N/D
Female with ICD-10 codes, by age group	15-39	90.7 (86.9-94.7)	N/D	92.8 (89.3-96.6)	96.6 (93.8-99.4)	98.1 (95.9-100.1)	N/D	N/D
	40-49	89.2 (86.2-92.3)	N/D	90.7 (87.6-93.9)	93.3 (90.4-96.3)	96.0 (93.4-98.6)	N/D	N/D
	50-59	82.5 (79.2-85.9)	N/D	88.8 (85.6-92.2)	91.0 (87.7-94.4)	94.6 (91.6-97.8)	N/D	N/D
	60-69	75.5 (72.0-79.0)	N/D	83.3 (79.9-87.0)	90.0 (86.3-93.9)	93.9 (90.3-97.7)	N/D	N/D
	70-79	70.3 (65.3-75.7)	N/D	79.1 (73.4-85.3)	84.5 (78.6-90.9)	86.3 (77.2-96.5)	N/D	N/D

CIS, carcinoma in situ; CI, confidence interval; RS, relative survival; ICD-10, International Classification of Diseases, Tenth Revision; N/D, not determined due to insufficient follow-up time

Supplementary Table 8 5-year crude survival and 5-year age-standardized relative survival (ASR) by cancer type based on ICD-10 codes in the sensitivity analysis (diagnosed 2013-2022, excluding CIS cases)

Type of cancer	5-year crude survival (95% CI)	5-year ASR (95% CI)
all	76.3 (75.0-77.6)	74.9 (71.2-78.8)
stomach	71.5 (67.3-76.0)	72.7 (62.2-85.4)
colorectal	84.4 (81.8-87.0)	83.9 (76.5-92.2)
liver	36.5 (27.0-49.5)	41.3 (23.7-71.8)
lung	53.5 (48.4-59.2)	54.1 (41.6-71.4)
thyroid	99.2 (97.8-100.0)	N/D
leukemia	63.2 (52.6-76.0)	N/D
prostate	90.9 (87.1-94.9)	92.5 (83.9-101.1)
breast	93.1 (91.2-95.0)	96.3 (91.1-100.9)
cervix uteri	82.1 (75.2-89.6)	N/D
corpus uteri	85.7 (79.3-92.6)	N/D
ovary	75.5 (65.1-87.6)	N/D
oral	77.4 (68.9-86.9)	N/D
esophagus	54.7 (44.8-66.8)	N/D
biliary	34.7 (23.6-51.1)	22.5 (13.4-38.0)
pancreas	20.6 (13.8-30.8)	N/D
bladder	83.5 (76.3-91.4)	85.1 (70.0-100.4)
kidney	82.4 (76.1-89.3)	82.7 (68.3-99.7)
lymphoma	78.6 (72.3-85.4)	76.7 (62.5-94.2)
myeloma	44.0 (26.9-71.8)	N/D
brain	45.7 (30.2-69.0)	N/D
testis	100.0 (100.0-100.0)	N/D
skin	91.8 (85.5-98.7)	85.3 (69.3-103.3)
secondary cancer	17.6 (10.6-29.2)	N/D

CIS, carcinoma in situ; ICD-10, International Classification of Diseases, Tenth Revision; ASR, age-standardized relative survival; CI, confidence interval

N/D, not determined: ASR could not be estimated for some cancer types due to an insufficient number of survivors in certain age groups with adequate follow-up time.

Supplementary Table 9 5-year relative survival and age-standardized relative survival by cohort and period analyses among cancer survivors (including CIS cases)

5-year relative survival (95% CI)					
	Group	Cohort analysis, 2005-2011	Cohort analysis, 2012-2017	Period analysis, 2012-2016	Period analysis, 2017-2021
Male, by age group	15-39	79.4 (70.7-89.0)	87.4 (81.1-94.2)	88.6 (83.6-94.0)	86.9 (81.1-93.2)
	40-49	71.0 (62.1-81.2)	76.6 (70.7-82.9)	79.8 (74.5-85.3)	87.7 (83.7-91.9)
	50-59	68.5 (62.4-75.2)	77.0 (72.4-81.8)	80.0 (76.2-83.9)	81.8 (78.2-85.6)
	60-69	69.6 (63.0-76.9)	71.0 (66.7-75.6)	76.7 (72.7-80.8)	79.0 (75.5-82.7)
	70-79	54.3 (41.5-70.9)	70.0 (63.3-77.4)	71.6 (64.5-79.5)	74.1 (68.7-79.9)
Female, by age group	15-39	95.3 (92.1-98.6)	93.7 (91.0-96.5)	94.5 (92.3-96.8)	96.5 (94.7-98.3)
	40-49	88.6 (84.2-93.2)	89.4 (86.5-92.5)	90.4 (87.9-93.1)	93.5 (91.4-95.6)
	50-59	86.5 (81.9-91.3)	83.8 (80.2-87.5)	86.7 (83.8-89.7)	88.1 (85.4-91.0)
	60-69	77.5 (71.1-84.3)	75.0 (70.8-79.5)	78.5 (74.6-82.5)	81.6 (78.4-84.9)
	70-79	65.3 (53.0-80.5)	67.0 (60.9-73.7)	72.4 (66.1-79.2)	77.1 (72.4-82.2)
Age-standardized relative survival for all cancers combined	Overall	69.6 (61.8-79.6)	74.5 (70.3-79.0)	76.3 (72.2-80.8)	80.6 (77.3-84.1)
	Male	64.7 (53.0-82.3)	73.7 (67.2-81.1)	75.8 (69.5-83.0)	79.2 (74.0-85.0)
	Female	74.0 (64.4-87.7)	74.9 (69.4-81.1)	76.5 (71.1-82.7)	81.7 (77.3-86.5)

CIS, carcinoma in situ; CI, confidence interval

Supplementary Table 10 Incidence rates of second primary cancers or recurrences, as applicable, per 1,000 person-years among cancer-free survivors by sex, age group, and type of first cancer

	Group	Incidence rates (95% CI)			
		cancer-free 3-year survivors (first cancer diagnosed 2005-2019, including CIS cases)	cancer-free 4-year survivors (first cancer diagnosed 2005-2018, including CIS cases)	cancer-free 5-year survivors (first cancer diagnosed 2005-2017, including CIS cases)	Incidence rates for first cancers
Male, by age group	15-39	25.9 (15.8 - 40.0)	16.0 (7.7 - 29.4)	12.0 (4.4 - 26.1)	0.5 (0.5 - 0.6)
	40-49	16.3 (9.5 - 26.1)	13.5 (6.7 - 24.1)	16.0 (7.7 - 29.5)	2.6 (2.4 - 2.8)
	50-59	38.5 (30.2 - 48.3)	32.0 (23.6 - 42.5)	28.7 (19.8 - 40.4)	7.6 (7.2 - 8.1)
	60-69	56.8 (46.8 - 68.2)	46.9 (36.5 - 59.2)	50.4 (37.9 - 65.6)	17.8 (16.8 - 18.9)
	70-79	66.0 (47.9 - 88.6)	66.3 (44.1 - 95.8)	67.6 (40.0 - 106.8)	30.8 (28.1 - 33.7)
Female, by age group	15-39	18.0 (13.0 - 24.3)	13.4 (8.7 - 19.8)	12.9 (7.8 - 20.2)	1.8 (1.7 - 1.9)
	40-49	21.4 (16.3 - 27.6)	19.5 (14.1 - 26.4)	17.6 (11.8 - 25.3)	4.5 (4.3 - 4.8)
	50-59	21.5 (16.4 - 27.6)	19.5 (14.1 - 26.3)	17.5 (11.8 - 25.0)	6.1 (5.7 - 6.4)
	60-69	46.3 (37.1 - 57.0)	37.3 (27.9 - 49.0)	30.5 (20.7 - 43.3)	9.0 (8.5 - 9.6)
	70-79	31.2 (20.2 - 46.0)	23.2 (12.4 - 39.7)	23.1 (10.6 - 43.8)	12.3 (11.2 - 13.4)
By type of first cancer	stomach	30.1 (22.7 - 39.2)	27.9 (19.9 - 37.9)	28.9 (19.7 - 40.7)	N/A
	colorectal	24.7 (19.6 - 30.7)	20.1 (14.9 - 26.5)	19.3 (13.5 - 26.8)	N/A
	liver	202.4 (120.0 - 319.9)	131.9 (57.0 - 259.9)	142.6 (52.3 - 310.3)	N/A
	lung	71.3 (51.0 - 97.2)	61.6 (39.5 - 91.7)	40.6 (20.3 - 72.6)	N/A
	thyroid	34.0 (18.6 - 57.1)	31.0 (14.2 - 58.9)	30.5 (11.2 - 66.4)	N/A
	leukemia	34.2 (13.7 - 70.4)	12.4 (1.5 - 44.7)	15.7 (1.9 - 56.8)	N/A
	prostate/testis	30.6 (21.1 - 43.0)	25.7 (15.9 - 39.3)	24.6 (13.8 - 40.6)	N/A
	breast	25.0 (20.1 - 30.7)	22.2 (17.0 - 28.6)	19.8 (14.2 - 26.9)	N/A
	uterus	14.1 (9.9 - 19.4)	12.1 (7.8 - 17.8)	11.8 (7.1 - 18.4)	N/A
	ovary	53.9 (29.5 - 90.5)	40.2 (17.4 - 79.2)	32.3 (10.5 - 75.4)	N/A
	others	49.9 (42.3 - 58.4)	39.4 (31.9 - 48.2)	36.9 (28.6 - 46.9)	N/A

CI, confidence interval; CIS, carcinoma in situ; N/A, not applicable

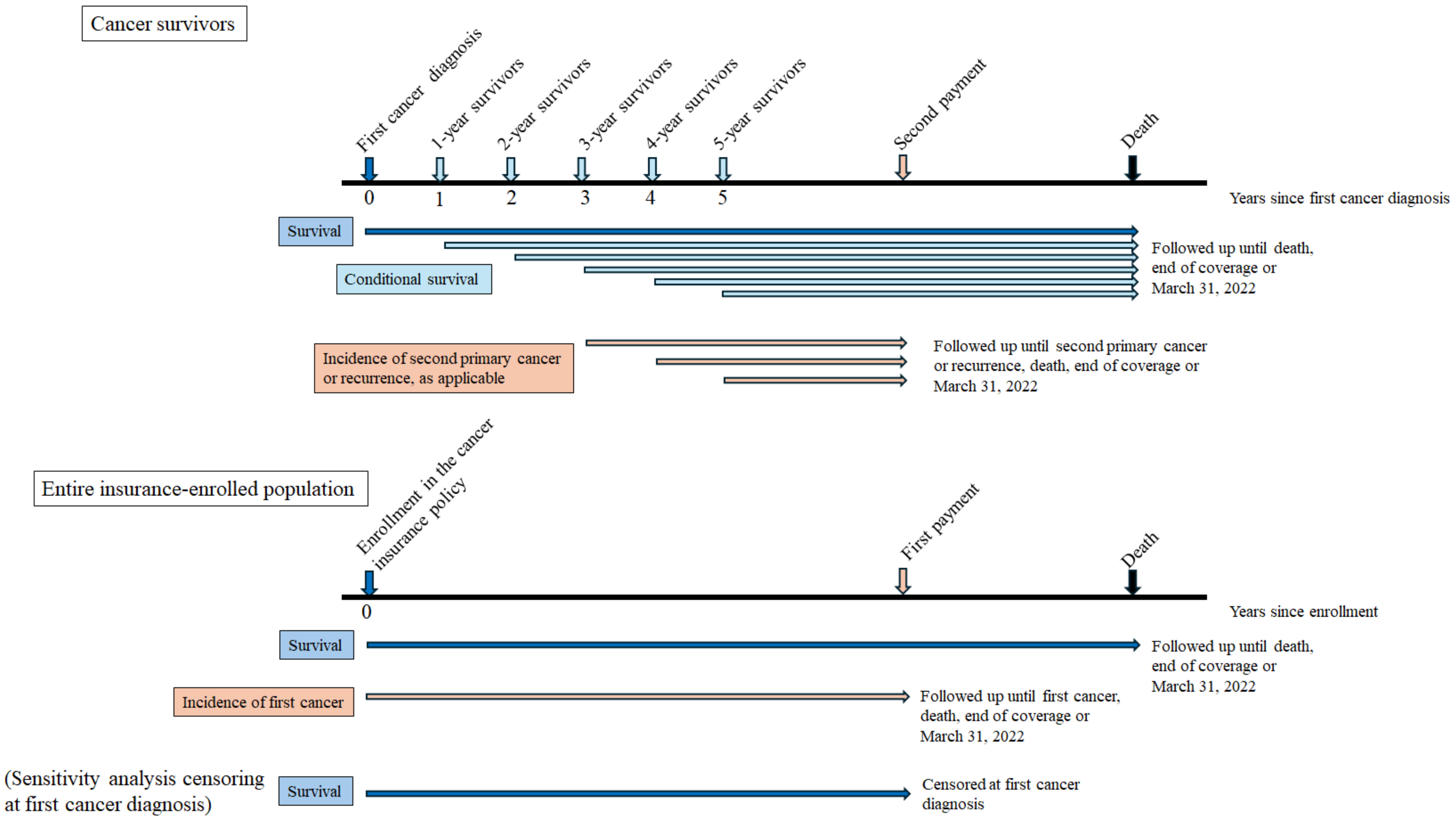

Supplementary Fig. 1 Definition of outcomes in the analyses of cancer survivors and the entire insurance-enrolled population

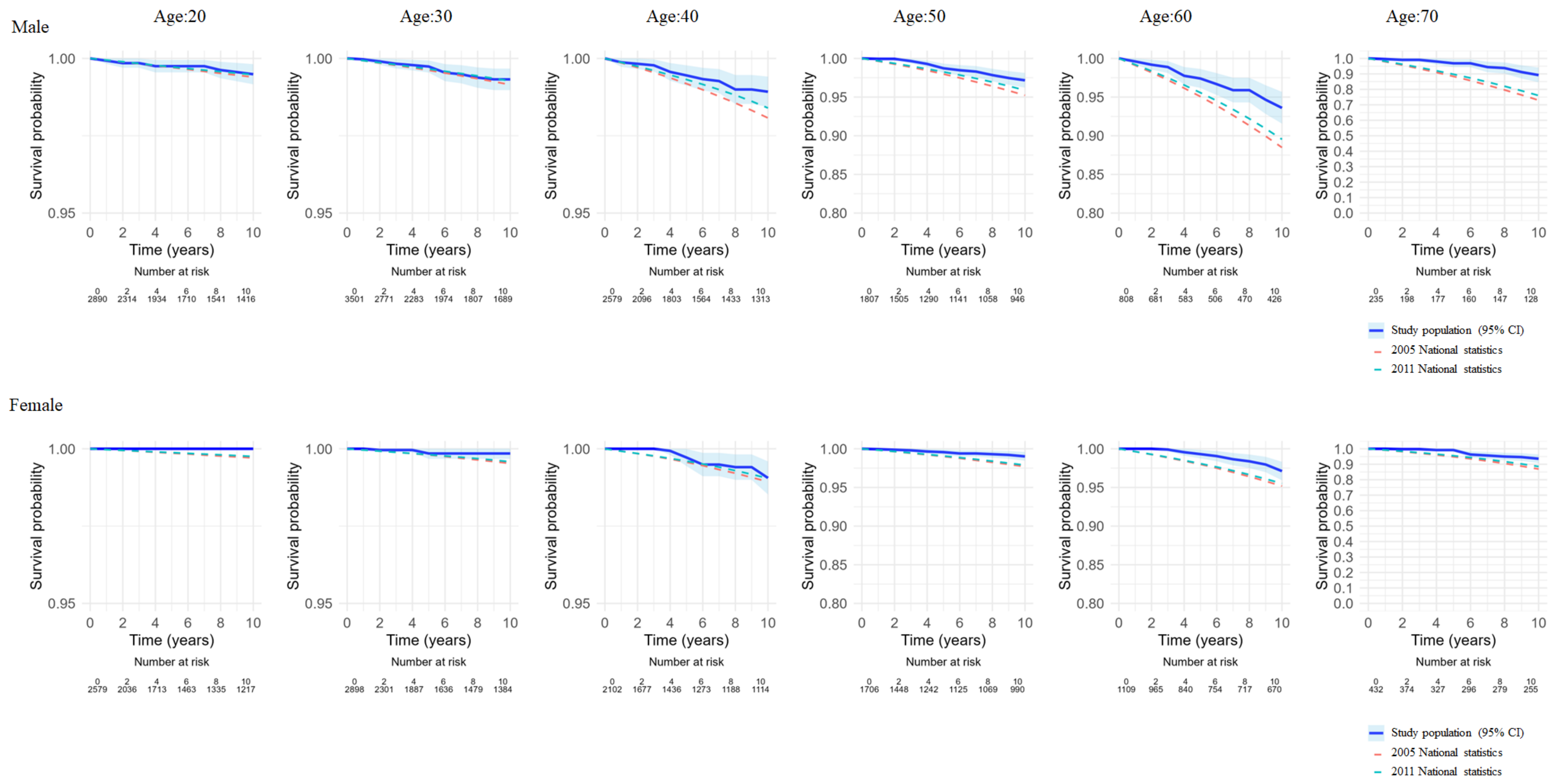

Supplementary Fig. 2 Cohort survival of the entire insurance-enrolled population in this study (year 2005-2011). CI, confidence interval

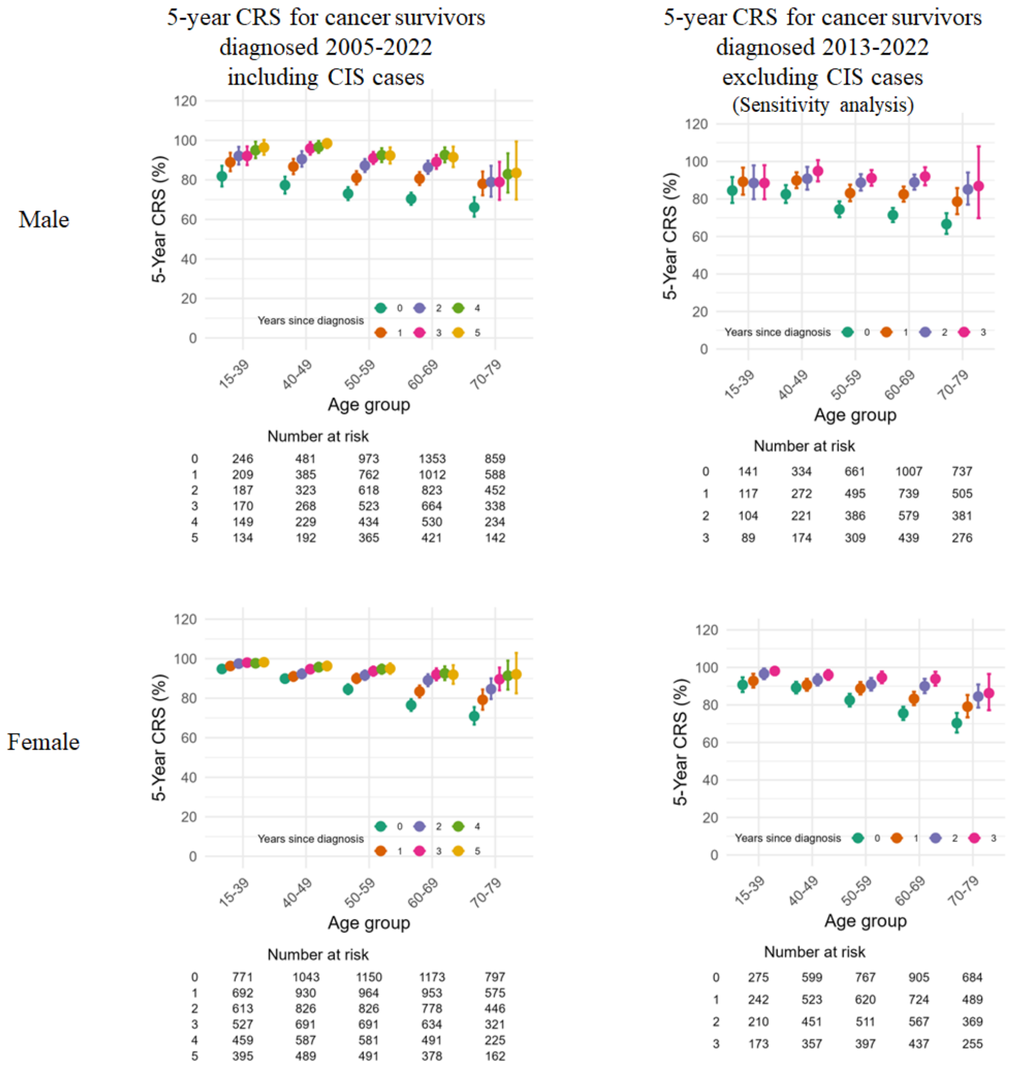

Supplementary Fig. 3 5-year conditional relative survival (CRS) among cancer survivors in the main analyses (including CIS cases) and sensitivity analyses (excluding CIS cases), with censoring at first cancer diagnosis in the reference population. Error bars indicate 95% confidence intervals. CIS, carcinoma in situ

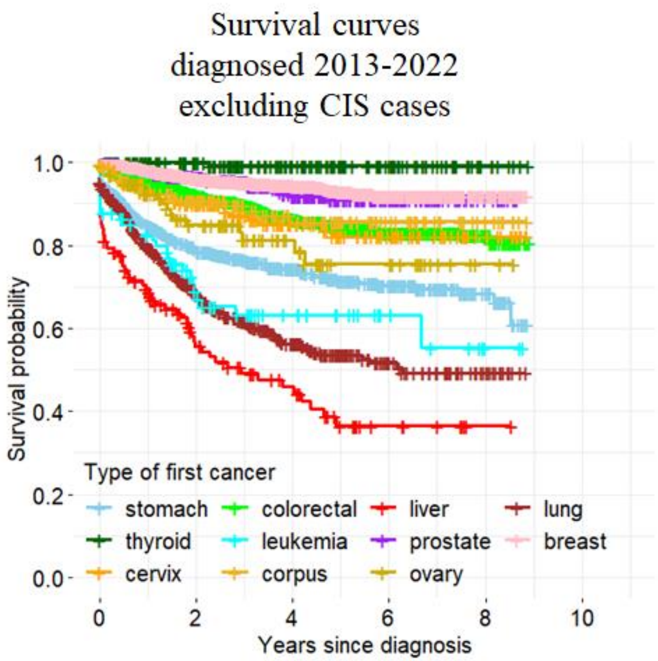

Number at risk

Type of first cancer	0	2	4	6	8	10
stomach	568	339	205	117	40	0
colorectal	1205	844	458	223	59	0
liver	121	47	28	8	1	0
lung	520	251	121	45	14	0
thyroid	185	133	84	43	7	0
leukemia	82	39	22	9	2	0
prostate	399	262	144	62	12	0
breast	1051	782	485	237	64	0
cervix	157	119	67	38	11	0
corpus	149	103	59	29	10	0
ovary	97	60	30	16	3	0

Years since diagnosis

Supplementary Fig. 4 Survival curves by type of first cancer in the sensitivity analysis (excluding CIS cases). CIS, carcinoma in situ

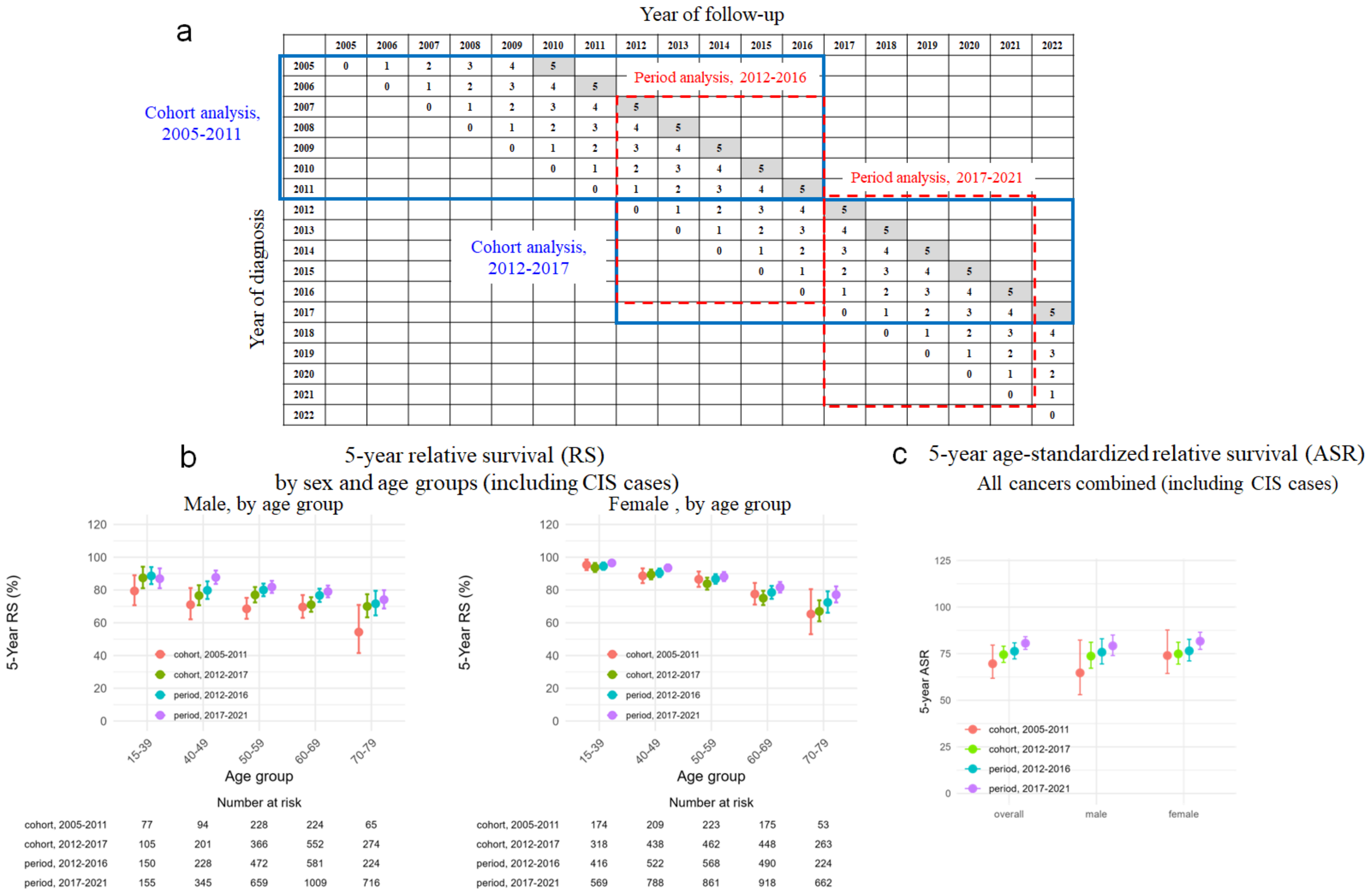

Supplementary Fig. 5 **(a)** Illustration of time scales in cohort and period analyses (b) 5-year relative survival by sex and age group, and **(c)** 5-year age-standardized relative survival for all cancers (including CIS cases) combined in cohort and period analyses. Error bars indicate 95% confidence intervals. CIS, carcinoma in situ

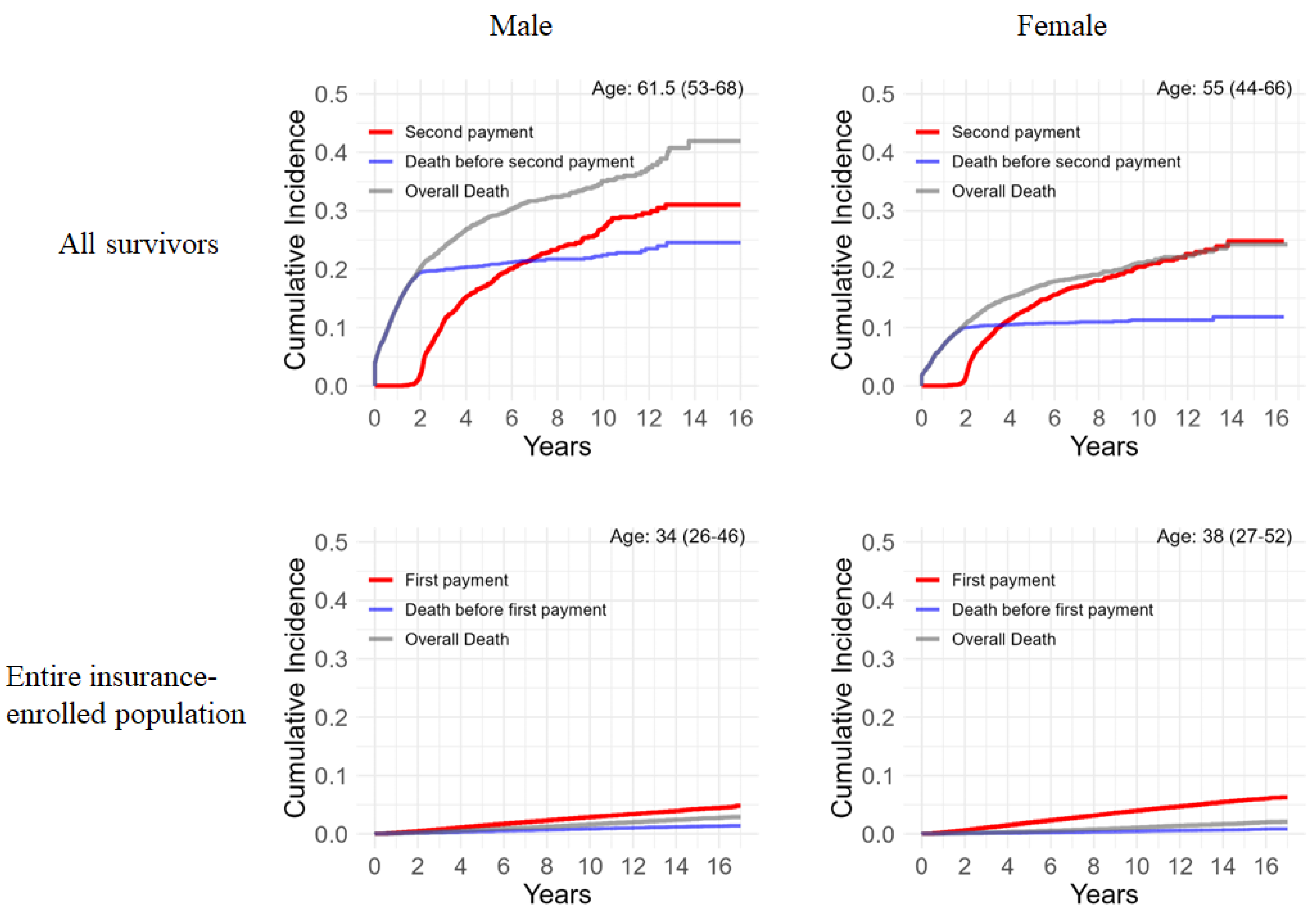

Supplementary Fig. 6 Cumulative incidence of cancer, death before cancer and overall death among cancer survivors (diagnosed 2005-2022, including CIS cases) and the entire insurance-enrolled population. CIS, carcinoma in situ
